# Metastases and treatment-resistant lineages in patient-derived cancer cells of colorectal cancer

**DOI:** 10.1038/s42003-023-05562-y

**Published:** 2023-11-24

**Authors:** Shiki Fujino, Norikatsu Miyoshi, Aya Ito, Rie Hayashi, Masayoshi Yasui, Chu Matsuda, Masayuki Ohue, Masafumi Horie, Shinichi Yachida, Jun Koseki, Teppei Shimamura, Tsuyoshi Hata, Takayuki Ogino, Hidekazu Takahashi, Mamoru Uemura, Tsunekazu Mizushima, Yuichiro Doki, Hidetoshi Eguchi

**Affiliations:** 1 Department of Gastroenterology, Central Clinical School, Monash University, Melbourne, VIC Australia; 2 Department of Gastroenterological Surgery, Osaka University Graduate School of Medicine, Suita-City, Osaka Japan; 3 Innovative Oncology Research and Translational Medicine, Osaka International Cancer Institute, Chuo-ku, Osaka, Japan; 4 Department of Surgery, Osaka International Cancer Institute, Chuo-ku, Osaka, 541-8567 Japan; 5 Department of Cancer Genome Informatics, Osaka University Graduate School of Medicine, Suita-City, Osaka Japan; 6 Division of Systems Biology, Graduate School of Medicine, Nagoya University, Nagoya-City, Aichi, Japan

**Keywords:** Colorectal cancer, Tumour biomarkers

## Abstract

Circulating tumor cells (CTCs) play an important role in metastasis and recurrence. However, which cells comprise the complex tumor lineages in recurrence and are key in metastasis are unknown in colorectal cancer (CRC). CRC with high expression of POU5F1 has a poor prognosis with a high incidence of liver metastatic recurrence. We aim to reveal the key cells promoting metastasis and identify treatment-resistant lineages with established EGFP-expressing organoids in two-dimensional culture (2DOs) under the *POU5F1* promotor. POU5F1-expressing cells are highly present in relapsed clinical patients’ blood as CTCs. Sorted POU5F1-expressing cells from 2DOs have cancer stem cell abilities and abundantly form liver metastases in vivo. Single-cell RNA sequencing of 2DOs identifies heterogeneous populations derived from POU5F1-expressing cells and the Wnt signaling pathway is enriched in POU5F1-expressing cells. Characteristic high expression of *CTLA4* is observed in POU5F1-expressing cells and immunocytochemistry confirms the co-expression of POU5F1 and CTLA4. Demethylation in some CpG islands at the transcriptional start sites of *POU5F1* and *CTLA4* is observed. The Wnt/β-catenin pathway inhibitor, XAV939, prevents the adhesion and survival of POU5F1-expressing cells in vitro. Early administration of XAV939 also completely inhibits liver metastasis induced by POU5F1-positive cells.

## Abstract (web summary)

A small population POU5F1-positive circulating tumor cells (CTC) have cancer stem cell abilities and contribute to colorectal cancer aggressiveness and metastasis.

## Introduction

Cancer is a leading cause of death globally due to its uncontrolled proliferative and metastatic potential^1^. In colorectal cancer (CRC), some patients respond well to conventional treatments; however, most advanced tumors (refractory cancers) recur despite an initially good response to therapy, resulting in a poor prognosis^2^. Although the mechanisms underlying treatment resistance in CRC have been partially elucidated^3^, based on characteristic genes and the response to specific treatment strategies^4^, several cancers remain refractory because of heterogeneity caused by genetic and epigenetic diversity^5^.

Circulating tumor cells (CTCs), which play critical roles in recurrence and distant metastasis^6^, and several CTC markers have been reported in different types of cancers^7^. In CRC, mesenchymal markers, PI3Ka, CEA, and PRL3, were reported as CTC markers; however, no marker has been established for clinical use. Furthermore, tumor heterogeneity in CRC is thought to be promoted by small cancer stem cell (CSC) populations, as well as regulatory genes such as *LGR5*^8^, which encode important factors in treatment resistance^5^. Surprisingly, CRCs retain the same histological morphology in recurrent and metastatic lesions and do not undergo dedifferentiation, although differences exist in gene expression and signaling pathway activation^9^. Various lineages of CSCs induce cell differentiation and are thought to be important for metastasis. However, CTCs of CRC were recently reported as *LGR5-*negative^10^.

POU5F1 (OCT4) is a stem cell factor in embryogenesis and its increased expression is related to chemotherapy resistance in several cancers^11^. We also reported POU5F1’s role in the development of chemoresistance via HNF1A expression in CRC cells^12^. POU5F1 was reported to be a prognostic CTC marker in glioblastoma^13^ and bladder cancer^14^; however, the direct role of POU5F1-expressing cells in distant metastasis is mostly unknown.

Herein, we aimed to identify POU5F1-positive cells involved in tumor heterogeneity and treatment resistance and explore their mechanism of metastasis using organoids in two-dimensional culture (2DOs). Primary cultured cancer cells are an excellent model for unraveling the mechanisms of tumor diversity^15^ and can help reproduce clinical therapeutic resistance^16,17^. Additionally, we aimed to characterize the resistant population produced from POU5F1-positive cells to identify new therapeutic targets by single-cell RNA sequencing analysis.

## Results

### POU5F1-expressing cells were identified in clinical blood samples

**Fig. 1:**
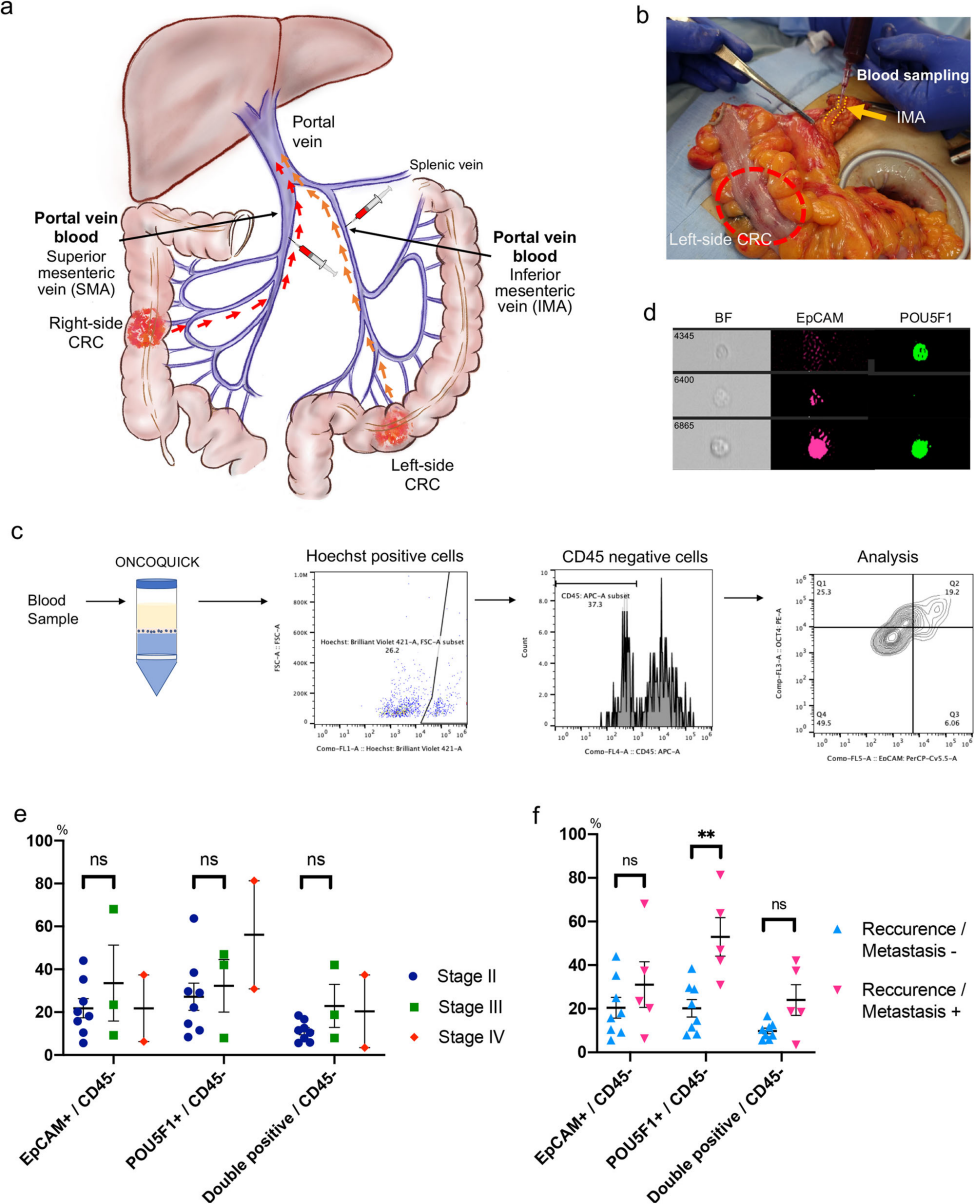
Analysis of POU5F1-positive cells in clinical blood samples. **a** Schematic representation of blood sampling in a clinical specimen for circulating tumor cell analysis. **b** Representative image of blood sampling. **c** Schematic representation of the preparation of collected blood and fluorescence-activated cell sorting analysis. **d** Representative morphology of analyzed cells. **e** The ratio of EpCAM-positive and POU5F1-positive cells to CD45-negative cells in blood, according to TNM stage. (mean value ± SEM). **f** The ratio of EpCAM-positive and POU5F1-positive cells to CD45-negative cells in blood according to recurrence and metastasis. (**P < 0.01, mean value ± SEM) *BF* Bright figure; *CRC* colorectal cancer; *n.s.* not significant.

The presence of POU5F1-expressing cells was examined in clinical specimens. Blood samples were collected from the superior and mesenteric veins in right-sided and left-sided CRC, respectively, obtained during surgery as an alternative to portal vein samples (Fig. 1a, b). After ONCOQUICK preparation, Hoechst-positive and CD45-negative cells were analyzed as circulating cells (Fig. 1c). Among these, EpCAM-expressing and POU5F1-expressing cells were evaluated. The morphology was visually confirmed, and cells analyzed as CTCs maintained their cell morphology (Fig. 1d). Eight samples from stage II patients, three from stage III, and two from stage IV (according to the TNM stage^18^) were examined. There was no significant difference in the ratio of EpCAM-expressing cells and POU5F1-expressing cells to CD45-negative cells between stage II and stage III patients (Fig. 1e). No statistical analysis was performed in stage IV patients due to the small sample size. The relationship between the ratio and recurrence/metastasis was then examined (median follow-up period: 2.4 years, range: 1.1–3.1 years). Patients with recurrence after surgery and stage IV patients with metastases at the surgery were included in the recurrence/metastasis group. EpCAM-expressing cells did not appear to differ between the two groups; however, the ratio of POU5F1-expressing cells was higher in the recurrence/metastasis group than in the non-recurrence/metastasis group (Fig. 1f). Thus, the significance of POU5F1-expressing cells as CTCs may be different from EpCAM-expressing cells, suggesting that they may be involved in the recurrence/metastasis of CRC.

### Establishment of 2DOs reflected clinical malignancy

**Fig. 2:**
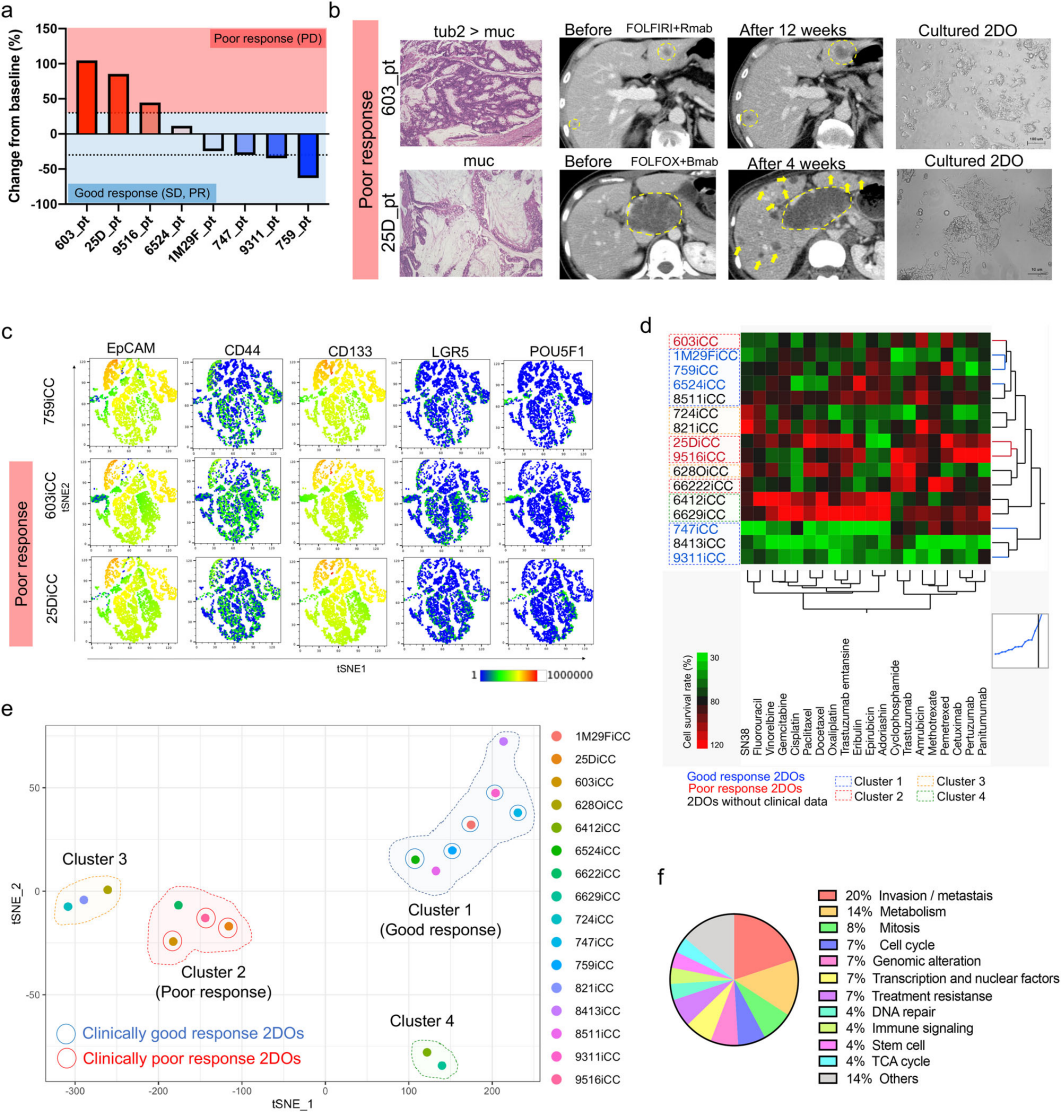
2DOs reflect resistance to clinical treatment. **a** Change in target lesion size from baseline for each patient. **b** Representative histological and computed tomography images of patients with two poor responses and microscopic images of cultured 2DOs. Scale: 100 μm. **c** Expression of evaluated stem cell markers (CD44, CD133, and LGR5), EpCAM, and POU5F1 in several 2DOs (759iCCC, 603iCC, and 25DiCC) by fluorescence activated cell sorting analysis. **d** Heat map and two-dimensional clustering of the cell viability of 16 2DOs after anticancer drug treatment (average of three independent tests). Red, blue, and black represent 2DOs with poor response, good response, and no clinical data, respectively. The dotted squares indicate clusters 1–4 in (**e**). **e** Drug responsiveness by dimensional compression of cell viability for all drugs tested. Clusters 1 and 2 contained 2DOs with clinically good and poor responses, respectively. Clusters 3 and 4 could not be determined because of lack of clinical data. **f** Functional classification of the top 100 enriched gene sets in poor-response 2DOs. 2DO organoid in two-dimensional culture.

2DOs were established from surgically resected tumors identified as clinically highly refractory and highly malignant for further analysis of POU5F1-expressing cells. The treatment response rates in eight patients with CRC and distant metastasis are shown in Fig. 2a and details of the respective treatment regimens are shown in Supplementary Table S1. Patients confirmed to have progressive disease according to the RECIST criteria^19^ were included in the poor-response group, whereas patients with stable disease and partial response were included in the good response group. No patient showed a complete response. Representative computed tomography, histological images, and cultured 2DOs of two poor-response patients are shown in Fig. 2b. 2DOs derived from primary CRC specimens that maintained clinical tumor heterogeneity and differentiation status were cultured and examined according to previous reports^12,20^. Flow cytometric analysis showed that the population of POU5F1-positive cells was also detected in 2DOs as cancer cells and differed from previously reported CRC stem cell marker-positive cells, including those with the markers, CD133, CD44, CD24, and LGR5^21^ (Fig. 2c). Drug sensitivities of sixteen 2DOs, including eight 2DOs derived from patients with distant metastases as mentioned above, were evaluated (Fig. 2d). Analysis by *t*-SNE clearly showed four clusters, including two clusters based on the response to therapy (good and poor) (Fig. 2e). RNA sequencing also confirmed that, compared with good-response 2DOs, poor-response 2DOs were enriched in gene sets related to distant metastasis, recurrence, and treatment resistance (Fig. 2f, Supplementary Data 1). Therefore, further analyses were performed using mainly 603iCC and 25DiCC, the top two 2DOS that were clinically less effective in suppressing tumors with chemotherapy.

### POU5F1-positive cells have CTC characteristics and are drug resistant

**Fig. 3:**
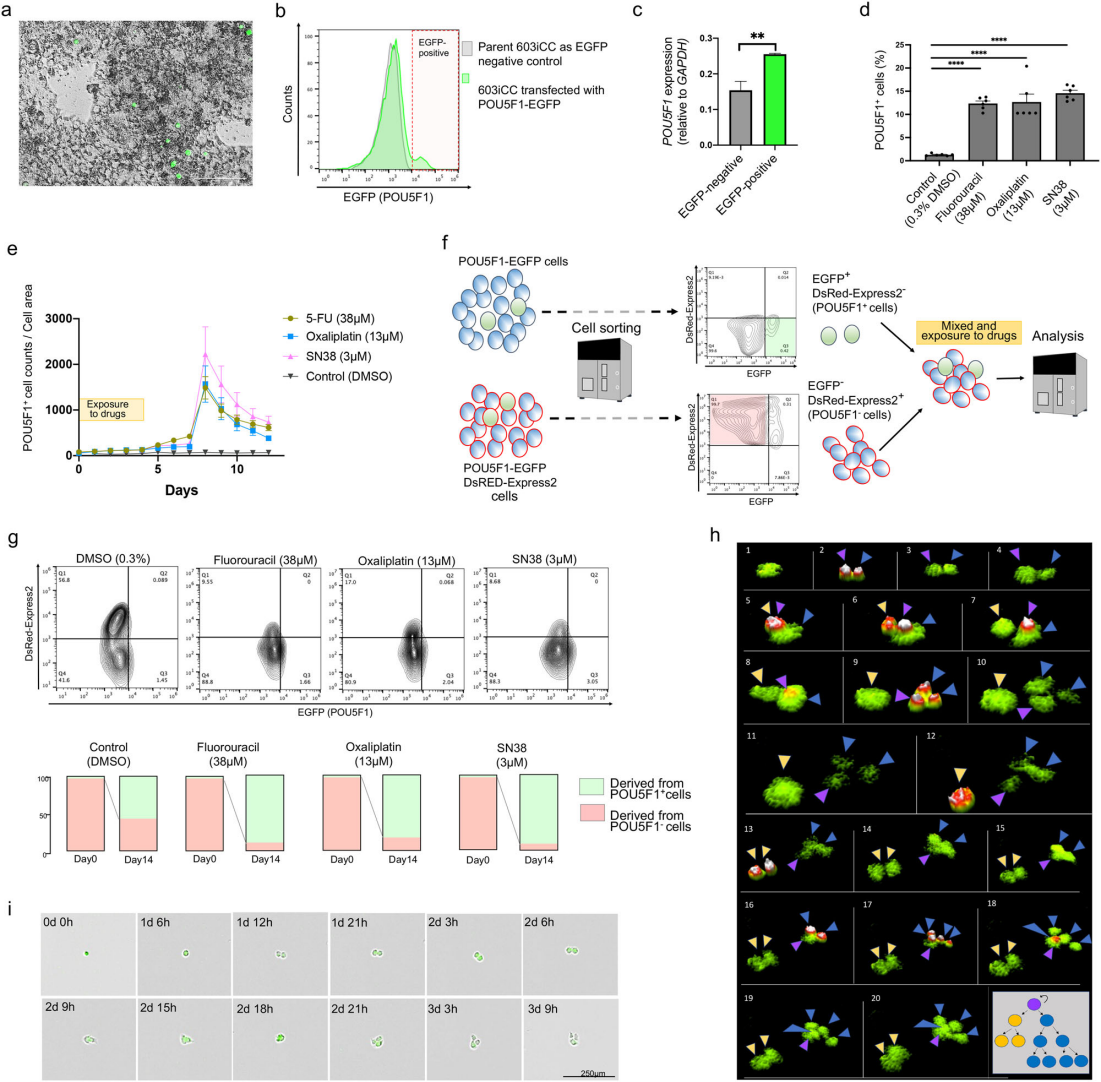
POU5F1-positive cells were resistant to treatment and caused tumor heterogeneity. **a** Representative phase contrast and EGFP fluorescence microscopy images of established and re-expanded 603iCC cells. Scale: 100 μm. **b** Flow cytometry analysis detected POU5F1(EGFP)-positive cells in established 603iCC cells. **c** POU5F1 expression in EGFP-negative and EGFP-positive cells by qRT-PCR (*n* = 3, **P < 0.01, mean value ± SEM). **d** Percentage of POU5F1-positive cells among remaining cells after anticancer drug treatment. (603iCC, *n* = 6, ****P < 0.001, mean value ± SEM). **e** Number of POU5F1-positive cells under anticancer drug administration and after discontinuation of anticancer drugs (603iCC, *n* = 4, median value ± SEM). **f** Schematic representation of the analysis of the origin of POU5F1-positive cells that proliferate after anticancer drug treatment. DsRed-Express2-expressing vector was transfected into POU5F1-EGFP cells. EGFP^+^ DsRed-Express2-negative cells were sorted (green area) and EGFP^-^ DsRed-Express2-positive cells were sorted (pink area). They were mixed and cultured at a ratio of 3:97 and exposed to anticancer drugs for 96 h (from day 0 to day 4). **g** Surviving cells (day 14) were analyzed by flow cytometry. POU5F1-positive cells were DsRed-Express2-negative. Most surviving cells (day 14) were derived from POU5F1-positive cells (603iCC, *n* = 4; median values are shown). **h** Representative images of asymmetric division; a “purple” cell produced “yellow” and “blue” cells, which in turn showed a symmetric division pattern in 603iCC. **i** Representative images of cell division. EGFP-negative cells were produced from EGFP-positive cells (603iCC). Scale: 250 μm.

2DOs were transfected with a vector expressing EGFP under the *POU5F1* promoter^22^ (Supplementary Fig. S1). When EGFP-positive cells regrew after sorting, many cells lost EGFP expression, and the proportion of EGFP-positive cells decreased to a few percent (Fig. 3a, b). qRT–PCR confirmed that cells that lost EGFP expression (EGFP-negative cells) had significantly lower *POU5F1* expression than EGFP-positive cells (Fig. 3c). In vitro administration of anticancer drugs to 2DOs induced cell death, but the proportion of POU5F1-positive cells was significantly higher among remaining cells in treated 2DOs than in untreated 2DOs (Fig. 3d). Interestingly, the number of POU5F1-positive cells increased significantly at the end of drug treatment, then subsequently decreased while the number of POU5F1-negative cells increased (Fig. 3e). In the DMSO control, there was no prominent increase in POU5F1-positive cells. Next, we examined whether the transient increase in POU5F1-positive cells after chemotherapy was a phenomenon of POU5F1-negative shifting into a POU5F1-positive state, or a case of POU5F1-positive cells dividing and proliferating. Some 2DOs expressing EGFP under the *POU5F1* promoter were double-transfected with a vector expressing DsRed-Express2. *POU5F1* expression in EGFP-positive and DsRed-Express2-positive cells was also confirmed by qRT–PCR (Supplementary Fig. S2). EGFP-positive cells without DsRed-Express2 (POU5F1-positive cells) and EGFP-negative cells with DsRed-Express2 (POU5F1-negative cells) were sorted and mixed at a ratio of 3:97, the ratio of the original population and those exposed to anticancer drugs (Fig. 3f). Fluorescence activated cell sorting (FACS) analysis revealed that cells in the regrowth population after chemotherapy, including EGFP-negative cells, were mostly derived from POU5F1-positive cells compared to that of the control, and all EGFP-positive cells were DsRed-Express2-negative (Fig. 3g). Thus, most of the cells remaining after treatment were produced from POU5F1-positive cells, and POU5F1-positive cells may play an important role in re-proliferation as key drug resistant cells. Furthermore, POU5F1-positive cells showed asymmetric division^23^ (Fig. 3h; Supplementary Movie S1); had characteristically a longer cell division time than POU5F1-negative cells (Supplementary Fig. S3a, b); and produced POU5F1-negative cells (Fig. 3i). Notably, singly isolated POU5F1-positive cells produced a heterogeneous population of cells expressing various differentiation markers, such as chromogranin A, cytokeratin 20 (CK20), mucin 2 (MUC2), and CD44, as stem cell markers from a single cell, both in vitro (Supplementary Fig. S4) and in vivo (Supplementary Fig. S5). Thus, POU5F1-positive cells could survive and re-construct heterogeneous cell populations with several differentiation stages, a characteristic of CSCs.

### POU5F1-expressing cells have a high capacity for forming metastases

**Fig. 4:**
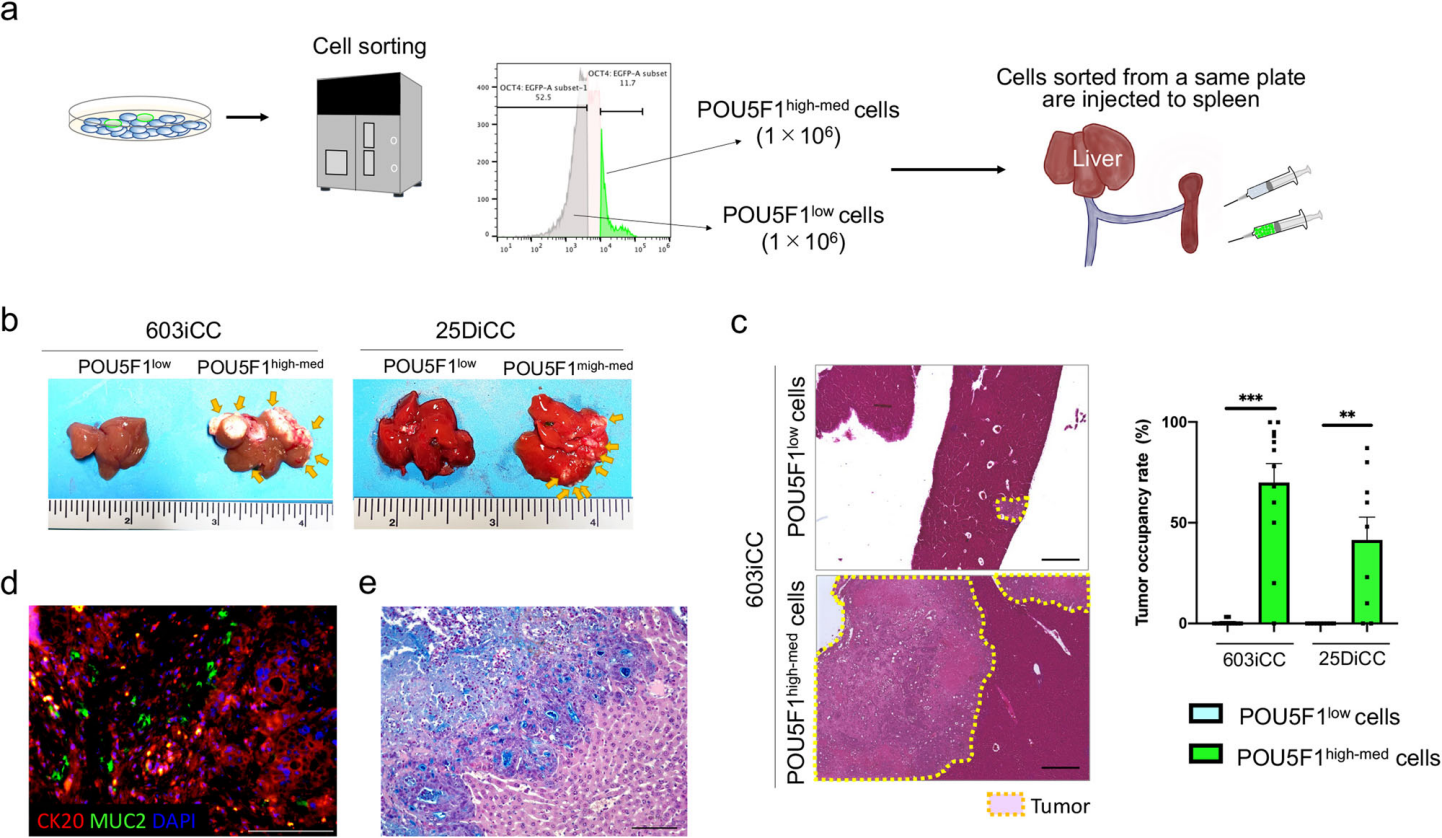
POU5F1-positive cells highly form heterogenic liver metastases. **a** Schematic representation of POU5F1^high-med^ and POU5F1^low^ cell sorting and the creation of a liver metastasis model by splenic injection. **b** Representative macroscopic images of the liver following intra-splenic administration of POU5F1^high-med^ and POU5F1^low^ cells from two 2DOs (603iCC and 25DiCC). **c** Evaluation of the proportion of liver metastases using H&E staining. The tumor occupancy rates of every 1-mm liver lobe section were measured (603iCC, *n* = 12; 25DiCC, *n* = 9; **P < 0.01, ***P < 0.001, mean value ± SEM). Scale: 500 μm. **d** Immunohistochemistry of CK20 and MUC2 in an in vivo tumor formed by POU5F1^high-med^ cells. Scale: 100 μm. **e** Alcian blue staining of an in vivo tumor formed by POU5F1^high-med^ cells. Scale: 100 μm. 2DO, organoid in two-dimensional culture.

Cells were sorted according to POU5F1 expression and injected into the spleen of mice to examine their ability to form liver metastases. The high-expressing population (high), labeled as positive, and intermediate (med)-expressing sorted cells were used to ensure there were enough cells for transplantation into the mice (Fig. 4a). Even if they were derived from the same 2DO, none of the POU5F1^low^ cells metastasized macroscopically (0%, 0/3 cases in 603iCC, 0/4 cases in 25DiCC), whereas the POU5F1^high-med^ cells formed metastases at a significantly higher rate (86%, 3/3 cases in 603iCC and 3/4 cases in 25DiCC) (*P* = 0.014 and *P* = 0.029) (Fig. 4b; Supplementary Fig. S6). Microscopic evaluation of the tumor occupancy rate also revealed a higher rate with POU5F1^high-med^ cells than with POU5F1^low^ cells (Fig. 4c). Liver metastases made from POU5F1^high-med^ cells produced cells positive for the differentiation markers, CK20 and MUC2 (Fig. 4d), and produced mucus (Fig. 4e), recapitulating tumor differentiation.

### POU5F1-positive cells induce treatment resistance through the Wnt pathway and CTLA4 expression

**Fig. 5:**
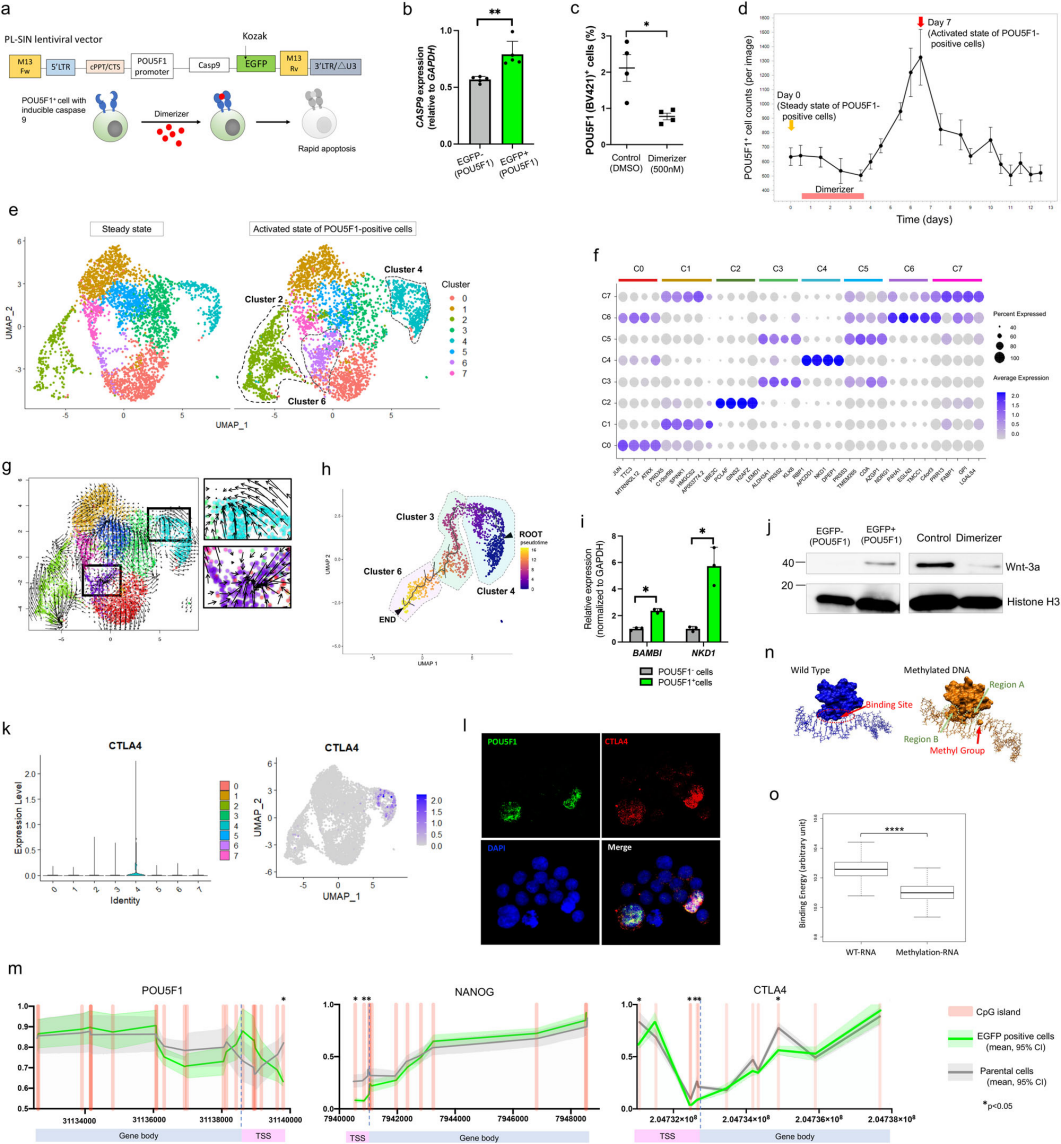
Single-cell RNA sequencing of treatment-resistant cells induced from POU5F1-positive cells. **a** Schematic of a new vector designed to induce apoptosis in cells expressing POU5F1 upon exposure to a dimerizer. **b** *CASP9* expression in EGFP-negative and EGFP-positive cells by qRT-PCR (603iCC, *n* = 5, **P < 0.01, mean value ± SEM). **c** Decreases in the number of POU5F1-positive cells by the dimerizer was determined by anti-POU5F1 antibody using flow cytometry (603iCC, *n* = 4, *P < 0.05, mean value ± SEM). **d** After 96 h of 500 nM dimerizer administration, the number of EGFP-positive cells surged, whereas their proportion decreased with cell proliferation (603iCC, *n* = 5, median value ± SEM). **e** Clustering of gene expression in single cells from 603iCC in steady and activated states of POU5F1-positive cells after treatment. **f** Characteristic genes in each cluster. **g** RNA velocity analysis. **h** Representative single-cell trajectory analysis starting from the expression of EGFP. **i** *BAMBI and NKD1* expression in EGFP-negative and EGFP-positive cells by qRT-PCR (603iCC, *n* = 3, *P < 0.05, mean value ± SEM). **j** Wnt-3a expression in EGFP-negative cells, EGFP-positive cells, cells treated with 0.3% DMSO (as control) for 96 h, and cells treated with 500 nM dimerizer for 96 h by western blotting. (603iCC). **k** Expression of *CTLA4* in each cluster. **l** Representative immunocytochemistry images of POU5F1 and CLTA4 staining of cells in vitro (603iCC). **m** Methylation of *POU5F1*, *NANOG, and CTLA4* in EGFP-positive and parental 603iCC on known CpG islands. **n** Changes in NANOG binding at the methylated TSS of *POU5F1*. Differences in representative binding configurations between wild-type and methylated DNA sequences at the first CpG island of *POU5F1*. **o** The binding energy distributions between NANOG protein and each DNA. Higher values indicate stronger binding energy. The addition of a methyl group to cytosine in the CpG island tended to reduce the DNA-binding energy for NANOG (****P < 0.001, mean value ± SEM).

A new vector was constructed to control POU5F1-positive cells. To specifically induce apoptosis in POU5F1-positive cells after dimerizer administration, cells were transfected with a vector in which Caspase-9 expression was induced by the *POU5F1* promoter (Fig. 5a). qRT–PCR confirmed that EGFP-positive cells had significantly higher *CASP9* expression than EGFP-negative cells (Fig. 5b), and *POU5F1* expression and *CASP9* expression were reduced by dimerizer (Supplementary Fig. S7). Dimerizer-induced reduction of POU5F1-positive cells was also confirmed by FACS analysis using anti-POU5F1 antibody (Fig. 5c). POU5F1-positive cells were suppressed in a concentration-dependent manner upon dimerizer administration (Supplementary Fig. S8). Dramatic POU5F1-positive cell proliferation was observed after weak suppression of POU5F1-positive cells using a small amount of dimerizer (Fig. 5d). This phenomenon was also observed after anticancer drug administration, suggesting that stress on stem cells due to treatment or other factors may have triggered the activity. By defining the mechanism of CSC activation, it was speculated that resistance to clinical treatment, such as recurrence with cell heterogeneity, could be elucidated^24^.

Single-cell analysis was performed to identify the differentiation lineages of POU5F1-positive cells. Overall, 8,248 single cells had RNA data that passed stringent quality control. The mean number of reads per cell was 101,989 and the median number of identified genes was 5,679. Eight characteristic clusters were identified (Fig. 5e) and their specific genes are shown in Fig. 5f and Supplementary Data 2. The number of cells in clusters 2, 4, and 6 was increased in the activated state of POU5F1-positive cells (Supplementary Fig. S9). Cluster 2 was characterized by the cell-cycle score (Supplementary Fig. S10), and the flow in RNA velocity seemed to advance toward the upper left of cluster 4 and around the center of cluster 6 (Fig. 5g). Since an analysis based on *POU5F1* expression was difficult due to its low expression value, *EGFP* expression was used as an alternative value (Supplementary Fig. S11). When cluster 4 (high *EGFP* expression) was set as the root, the proliferative trajectory of clusters 4 → 3 → 6 emerged, with characteristic gene expression profiles (Fig. 5h; Supplementary Figs. S12–14). Clear fluctuations in *EGFP* expression were observed, suggesting a process of differentiation originating from POU5F1-positive cells and cluster 4 had high *CLU* expression (Supplementary Fig. S15). *LGR5* expression was also observed in these clusters. We focused on cluster 4 as a starting point for POU5F1-positive cells and found that the Wnt signaling pathway was enriched (Supplementary Fig. S16), supporting stem cell properties^25^. qPCR confirmed increased gene expression associated with the Wnt pathway detected by pseudo time analysis in POU5F1-positive cells (Fig. 5i). Western blotting also confirmed the increased expression of Wnt-3a in the POU5F1-positive cells and that was decreased by dimerizer (Fig. 5j, Supplementary Figs. S17,S18). Glycolysis, HIF-1 signaling, and ferroptosis pathways, reported as properties of persister cells^26^, were enriched in cluster 6, located downstream of cluster 4, suggesting that these are produced by POU5F1-positive cells (Supplementary Fig. S19). POU5F1-positive cells remained after treatment; likewise, various treatment-resistant cells were produced from activated POU5F1-positive cells. Accordingly, we investigated how POU5F1-positive cells could escape the immune system and be present in the blood as CTCs. The expression of immune-related genes is shown in Supplementary Figure S20, and *CTLA4* was characteristically expressed in cluster 4 (Fig. 5k). Immunosuppressive molecules are important for cancer survival^27^, and CTLA4 allows CRC cells to escape from immunity^28^. Immunocytochemistry of the 2DO showed that POU5F1 and CTLA4 were co-expressed in the same cells (Fig. 5l). 2DOs were cultured in vitro for more than 1 year and therefore, this result meant that POU5F1-positive cells themselves produced CTLA4 and POU5F1-positive cells may be immune tolerant via CTLA4. Furthermore, methylation arrays revealed demethylation in some CpG islands at transcriptional start sites (TSSs) of *POU5F1, NANOG*, and *CTLA4* in EGFP-positive 603iCC 2DO cells compared to parental cells (Fig. 5m). NANOG forms a transcription network including pluripotency-regulating POU5F1^29^. Methylation of cytosine in CpG islands distorts the DNA double-helix structure, unlike the wild-type binding mode. The wild-type sequence is stably bound to the NANOG protein and does not distort the double-helix structure of the DNA. Conversely, when a methyl group is added to cytosine in the CpG island, the DNA cannot bind to the conventional region (Region A) and is forced to form a bond with a new region (Region B). As a result, it greatly distorts and destabilizes the double-helix structure of DNA. Accordingly, the estimated binding energy distribution for the methylated body shifts lower (Fig. 5n, o; Supplementary Fig. S21). Therefore, stress exerted on the cells by the treatment causes epigenetic changes and may regulate CSCs such as POU5F1-positive cells.

### Wnt inhibitor inhibits distant metastases formation by POU5F1-expressing cells

**Fig. 6:**
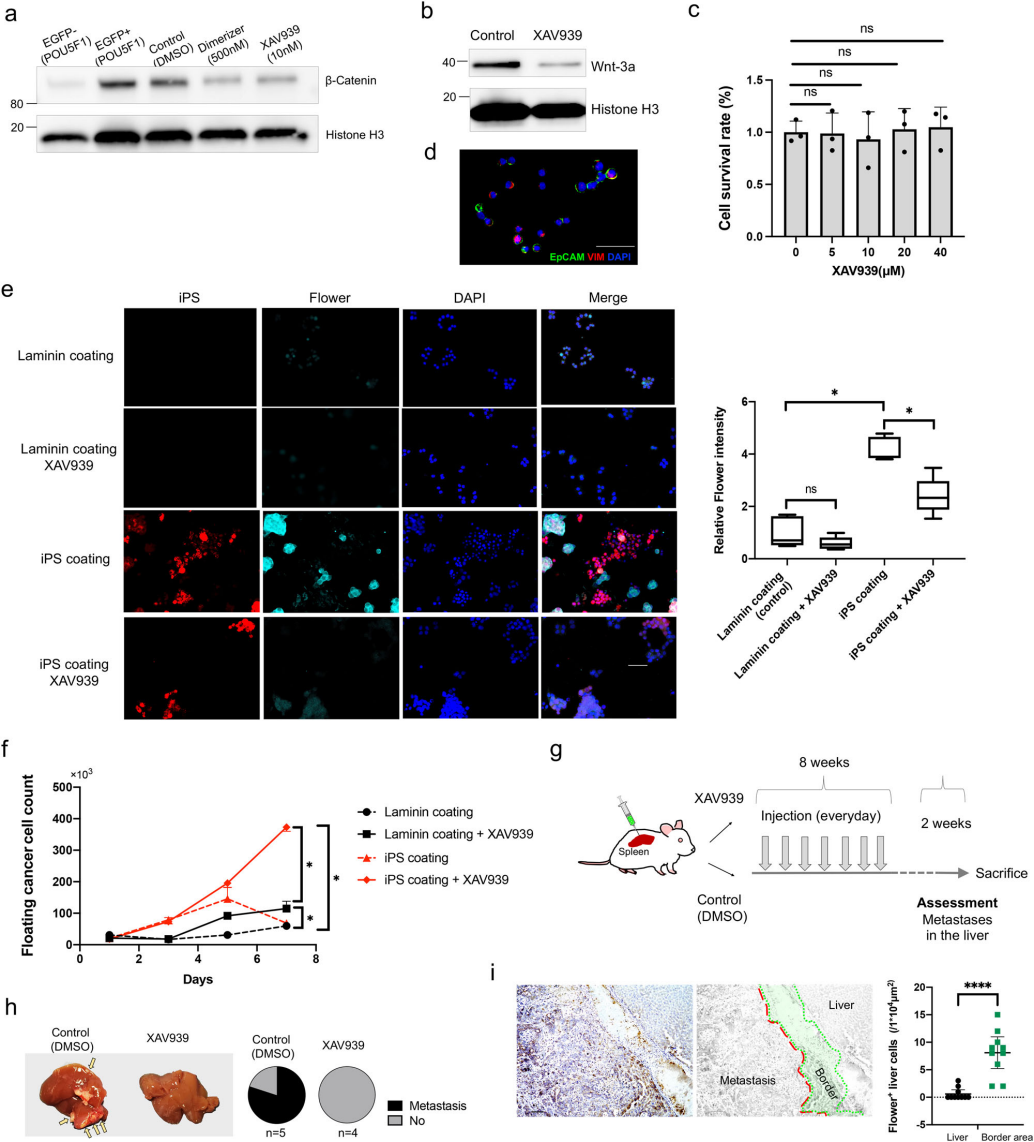
Wnt/β-catenin pathway inhibitor regulated POU5F1-positive cell liver metastasis. **a** β-Catenin expression in EGFP-negative cells, EGFP-positive cells, cells treated with DMSO (0.3%) for 96 h as control, cells treated with dimerizer (500 nM) for 96 h, and cells treated with XAV939 (10 μM) for 96 h by Western blotting. (603iCC). **b** Wnt-3a expression in cells treated with DMSO (0.3%) for 96 h as control, and cells treated with XAV939 (10 μM) for 6 h by Western blotting. (603iCC). **c** Cell proliferation rates after 96 h of exposure to XAV939 (603iCC, mean ± SEM. *n* = 3). **d** Representative immunocytochemistry of EpCAM and vimentin in vitro in iPS cells cultured in 2DO-culture medium. Scale: 50 μm. **e** Representative immunocytochemistry images of Flower in vitro in cells on laminin and iPS coatings, with and without XAV939 administration (603iCC, *n* = 5; *P < 0.05, mean ± SEM). **f** The number of floating cells on laminin and iPS coatings, with and without XAV939 administration (603iCC, *n* = 4; *P < 0.05, mean ± SEM). Scale: 50 μm. **g** Schematic representation of the evaluation of liver metastases, with and without XAV939. **h** Representative macroscopic image of the livers following intra-splenic administration of POU5F1-positive cells, with and without XAV939 administration. **i** Representative images and quantification of Flower immunohistochemistry in liver metastasis (603iCC without XAV939 administration, *n* = 10, ****P < 0.0001, mean ± SEM). Scale: 100 μm. 2DO organoid in two-dimensional culture; SEM standard error of the mean.

Single-cell trajectory analysis showed that the Wnt signaling pathway was enriched in cluster 4, originating from POU5F1-expressing cells. Western blot analysis confirmed increased β-Catenin expression in POU5F1-positive cells, which was reduced after dimerizer and Wnt inhibitor (XAV939) treatment (Fig. 6a, Supplementary Fig. S22, S23). Wnt-3a expression was also reduced by a Wnt inhibitor (Fig. 6b, Supplementary Fig. S24, S25). However, a normal proliferation assay did not show inhibition by a Wnt inhibitor in cell populations generated by POU5F1-positive cells (Fig. 6c). Niche cell-cell interactions are involved in adhesion and proliferation in the formation of clinical metastases^30^; therefore, we examined the relationship between a Wnt inhibitor and POU5F1-positive cells using a cell-cell interaction model. To mimic the metastatic niche, iPS cells were cultured in differentiation induction medium simulating the expression of both epithelial and mesenchymal markers (Fig. 6d). Compared to laminin coating of wells, iPS cell coating showed a significant increase in the expression of Flower protein, a cell-competitive marker^31,32^, which was significantly decreased by a Wnt inhibitor (Fig. 6e). Floating cancer cells (cells that failed to adhere and proliferate) were significantly increased by Wnt inhibitor treatment (Fig. 6f; Supplementary Fig. S26), suggesting that Wnt inhibitors may be able to inhibit the process of cell adhesion and proliferation, which are crucial processes in metastasis.

Finally, the inhibitory effect of a Wnt inhibitor on liver metastasis by POU5F1-expressing cells was examined in a mouse model. POU5F1-positive cells were sorted and injected into the spleen of mice to form liver metastases and a Wnt inhibitor was administered (Fig. 6g) In the control group, POU5F1-positive cells formed metastases at a significant high rate macroscopically (80%, 4/5 cases) (Fig. 6h) and Flower protein was also expressed in liver metastases and surrounding hepatocytes (Fig. 6i). In contrast, POU5F1-positive cells formed no metastases in the Wnt inhibitor group (0%, 0/4 cases).

## Discussion

We describe the role of POU5F1 in metastasis and resistance to clinical treatment in CRC. Our study found that POU5F1-positive cells produced heterogeneous populations from single cells even after treatment and were detected in clinical blood samples. Furthermore, we elucidated the partial role of POU5F1 in CRC by tracing its expression and identifying the involvement of POU5F1-expressing cells in treatment resistance. In particular, we firstly reported the finding that POU5F1-expressing cells express CTLA4, an immunosuppressive molecule.

CTLA4 is normally expressed on the surface of T-cells, suppressing the activation of T-cells^33^. Cancer cells induce immunosuppressive cells, such as Tregs expressing CTLA4, and suppress the action of dendritic cells via the CTLA4 pathway^34^. The significance of CTLA4 expression in CSCs remains unclear; however, CTLA4 on the CRC membrane contributes to the immunosuppressive microenvironment^28^ and helps CSCs survive. Our results suggest that POU5F1-positive cells could escape from the immune system by expressing CTLA4 and promoting metastases as CTCs.

Recently, it was reported that drugs could induce cells to enter a reversible drug-tolerant persister state, maintaining the clinical heterogeneity and clonal complexity of tumors in patient-derived xenograft models that recurred following treatment cessation^35^. We found that POU5F1-positive cells easily generated diverse cell clusters, each with typical characteristics (e.g., stem and persister cell characteristics), and the entire cell population was diverse and complex. Therefore, the ability to maintain complexity, rather than the characteristics of each cluster, should be considered an important factor in clinical recurrence and treatment. Furthermore, it has been reported that the drug-tolerant persister state exhibits significant transcriptional and functional similarities to the reversible state of diapause (interrupted embryonic development caused by adverse environmental conditions)^35^. Given that POU5F1 is also a relevant factor in embryogenesis^29^, our results are in line with earlier findings. Additionally, we found that *POU5F1* is an important gene that regulates stem cell characteristics and differentiation in CRC and is regulated by epigenetic changes. There are reports that anticancer drugs induce epigenetic changes^36^ and epigenetic reprogramming promotes cancer progression^37^. POU5F1 was reported to induce metabolic reprogramming in breast cancer^38^ and it may be a key molecule in CRC.

Lastly, we found that Wnt inhibitors completely inhibited metastasis induced by POU5F1-expressing cells. Wnt inhibitors have shown promise for stem cell suppression in a variety of carcinomas^39^. However, Wnt inhibitors have not yet reached clinical use due to their side effects, such as bone toxicity^40^. The present study indicated that POU5F1-positive cells show CRC stem cell characteristics and that inhibition of these cells by a Wnt inhibitor inhibits metastasis. Thus, the Wnt pathway is still an important therapeutic target, especially for CSCs. However, it should be noted that XAV939, as used in our experiment, does not directly inhibit Wnt3a, but functions by targeting tankyrase. XAV939 suppressed the expression of β-catenin and led to the suppression of Wnt3a expression in POU5F1-positive cells. A similar phenomenon was reported in a previous study^41^. Nevertheless, the underlying mechanism of action of Wnt in CSCs and its regulation remain unclear. Moreover, an important result from this study was that Wnt inhibition alone cannot suppress all the heterogeneous cell populations generated by POU5F1-positive cells. The timing of drug administration, the combination of drugs, and validation of these factors will be important in achieving clinical efficacy.

The present study has some limitations. Although the results suggest that CTLA4 may be important as an escape mechanism of POU5F1-positive cells in the human body, there were no confirmatory experiments. Thus, further experiments using humanized mice or using 2DOs and host immune cells and clinical data with large sample sizes are needed to confirm whether CTLA4 keeps CSCs alive and whether it is a key molecule. In addition, in the clinical sample analysis, the number of samples was not sufficient to analyze the relationship with recurrence, although the proportion of POU5F1-positive cells was predominantly higher in the combined recurrent and metastatic patient group. Some cells were thought to be epithelial-mesenchymal transition cells, expressing cell-surface vimentin; however, confirmation will be performed in a future analysis, and more cases and reanalysis are needed to verify the meaning of POU5F1-positive cells in blood. Moreover, we presented data showing that Wnt inhibitors are effective in suppressing POU5F1-positive cells, but we did not validate the numerous Wnt inhibitors or analyze differences in suppression by pathway. However, our study results suggest the importance of POU5F1-positive cells as CTCs, their escape mechanism, and a possible therapeutic approach. Floating of POU5F1-positive cells in the blood as CTCs may be prevented by inhibiting CTLA4; the adhesion and spread of POU5F1-positive cells to destination organs may be prevented by Wnt inhibitors. The identified molecules may be useful in the early stages of metastasis formation, rather than in the treatment of macro-metastasis. Further studies are needed to develop a related treatment that perfectly suppresses recurrence and metastasis.

## Conclusions

A 2DO model revealed that POU5F1-positive cells express an immunosuppressive molecule and promote clonal diversity, thereby supporting the proliferation of a refractory cell population, leading to therapeutic resistance and metastasis. The cells could be induced by epigenetic modifications, such as anticancer drug therapy. Furthermore, this cell population may be an important CTC forming distant metastases and Wnt inhibitors are key to their suppression.

## Methods

### Selection of patients and 2DOs

Eight patients with distant metastases or recurrences that could be evaluated using computed tomography were selected. All patients underwent baseline imaging within 4 weeks before anticancer drug administration. The tumor volume and reduction rate were measured according to RECIST guidelines^42^. 2DOs were established from primary CRC specimens and cultured according to a previous report^20^ and stocked at our laboratory cell bank. Briefly, CRC tissue from resected specimens was minced into 1-mm pieces and dissociated with 1 mg/mL of collagenase (C6885; Sigma-Aldrich, St. Louis, MO, USA). Filtered cell pellets between 20 μm and 200 μm were seeded in plates coated with iMatrix-511 (Takara Bio Inc., Kusatsu, Japan) and cultured in medium containing 10 ng/mL of basic fibroblast growth factor (ThermoFisher Scientific, Waltham, MA, USA) and 2 ng/mL of transforming growth factor beta (R&D Systems Inc., Minneapolis, MN, USA) to maintain heterogeneous primary culture cells. Sixteen 2DOs with stable culture and drug sensitivity on testing, including eight 2DOs from patients with distant metastases or recurrences, were selected for further analysis.

### Culture of CRC cell lines

The human colorectal tumor cell lines, HCT116, gifted by Dr. Bert Vongelstein (Johns Hopkins University, Baltimore, MD, USA), and HT29 (EC91072201, ECACC), were cultured in Dulbecco’s modified Eagle’s medium supplemented with 10% fetal bovine serum (ThermoFisher Scientific), 1% GlutaMAX‐I (ThermoFisher Scientific), and 1% penicillin/streptomycin/amphotericin B (Wako Pure Chemical Industries, Osaka, Japan). Cells were incubated at 37 °C in a humidified atmosphere containing 5% CO_2_. Cells were harvested using 0.25% Trypsin-EDTA (ThermoFisher Scientific) for further analysis.

### Culture of induced pluripotent stem (iPS) cell lines

Cellartis human iPS cell line 12 (ChiPSC12) cells (Takara Bio) were cultured in the Cellartis DEF-CS 500 Culture System (Takara Bio). Cells were incubated at 37 °C in a humidified atmosphere containing 5% CO_2_. Cells were harvested using Accutase (Innovative Cell Technologies, Inc., San Diego, CA) for further analysis.

### Flow cytometric analysis

The expression of proteins in cells was determined using flow cytometry. Cultured cells were dissociated with Accutase (Nacalai Tesque Inc., Kyoto, Japan). CTCs were isolated from clinical blood samples using OncoQuick (Greiner BioOne, Frickenhausen, Germany) according to the manufacturer’s protocol. Cells were stained with antibodies targeting EpCAM, CD133, CD44, CD41, CD45, and LGR5 (Supplementary Table S2). For detecting POU5F1, a True-Nuclear Transcription Factor Buffer Set (424401; BioLegend) was used. After staining cell surface proteins, cells were fixed and stained with antibodies for POU5F1, according to the manufacturer’s protocol. Relative fluorescent intensities were measured with an SH800 cell sorter (SONY, Tokyo, Japan) and cell morphology and staining locations were also measured with a FlowSight imaging flow cytometer (Merck-Millipore, Darmstadt, Germany). 7-AAD (Miltenyi Biotec, San Diego, CA, USA) was used to analyze living cells. A dimensionality reduction step in two dimensions was performed using *t*-distributed stochastic neighbor embedding (*t*-SNE) to visualize high-dimensional data of stem cell marker expression. Data were analyzed using FlowJo 10.2 software (FlowJo LLC, Ashland, OR, USA).

### Clinical anticancer drug sensitivity

Anticancer drug sensitivity was examined in sixteen 2DOs within 5–10 passages. Drugs and their concentrations in clinical drug assays are listed in Supplementary Table S3. The number of viable cells in each well was measured using a Cell Counting Kit-8 (Dojindo Laboratories, Kumamoto, Japan) before drug administration and 96 h after drug administration. Cell proliferation in DMSO and distilled water, which were used to dilute each drug, were used as controls. The ratio of the number of living cells after administering the drug to the control is shown. Three independent experiments were performed and the average is shown. The formula used for calculation was as follows: 100 × Cont. 0 h cell num. × Drug 96 h cell num./{(Cont. 96 h cell num.—Cont. 0 h cell num.) × Drug 0 h cell num.}

### Analysis of drug susceptibility by dimensional compression using t-SNE

Regarding the sensitivity of each anticancer drug, a dimensionality reduction step in two dimensions was performed using t-SNE to visualize high-dimensional data for 21 drugs in a low-dimensional space. The statistical analyses were performed using R 3.6.3 (R Core Team, 2018), with the data.table (v1.12.8; Dowle & Srinivasan^43^), t-SNE (Krijthe^44^), and ggplot2 (Wickham^45^) packages.

### RNA sequencing (RNA-seq) analysis of 2DOs

Total RNA was extracted using an RNA Purification Kit (Qiagen, Hilden, Germany). TruSeq Stranded mRNA Library Prep (Illumina, San Diego, CA, USA) was used to prepare RNA-seq libraries from the total RNA (1 µg). Multiplexed libraries were sequenced on an Illumina NextSeq with single-end 75-bp sequencing. RNA-seq data were mapped to the hg38 genome release using the bioinformatic pipeline of the Illumina Base Space Sequence Hub and the Subio software platform (Subio, Inc., Kagoshima, Japan).

### Establishment of OCT4 (POU5F1)-EGFP cells

The vector, PL-SIN-Oct4-EGFP, kindly provided by James Ellis (Addgene plasmid #21319; http://n2t.net/addgene:21319)^22^, was used to establish cells expressing EGFP under the *OCT4 (POU5F1)* promoter. The vector was transfected into 2DOs and cell lines using Lentiviral High Titer Packaging Mix with pLVSIN (Takara Bio). EGFP-positive cells were purified by sorting using a SH800 cell sorter (SONY) at least twice. *POU5F1* expression was confirmed by polymerase chain reaction (PCR).

### Quantitative real-time (qRT)-PCR analysis

Total RNA was isolated using an RNA Purification Kit (Qiagen). Quantitative assessment was performed by real-time PCR using 100 nM universal probe libraries, 0.1× FASTStart TaqMan Probe Master (Roche Diagnostics, Basel, Switzerland) for designed primers, iTaq Universal SYBR Green Supermix (Bio-Rad, Hercules, CA, USA) for commercially available primers, 100 nM primers, and 10 ng cDNA for cDNA amplification of target genes. Primers are listed in Supplementary Table S4. PCR was performed with 20 µL of the master mix in each well of a 96-well plate, and signals were detected with the CFX Connect Real-Time PCR Detection System (Bio-Rad). The thermocycler was programmed for one cycle at 95 °C for 10 min, followed by 40 cycles at 94 °C for 10 s, 60 °C for 20 s, and 72 °C for 1 s. cDNAs from NTERA-2 cells were used as positive controls.

### Xenograft model

A subcutaneous model was established to investigate the ability to differentiate from a single sorted cell. A single sorted cell was cultured in a dish for expansion using the 2DO culture methods described above. Accutane-dissociated cells (1 × 10^6^ cells) suspended in Matrigel (BD Biosciences, Franklin Lakes, NJ, USA) were subcutaneously transplanted into the dorsal flanks of 7-week-old, non-obese diabetic/severe combined immunodeficient mice (CLEA, Tokyo, Japan). The average weight was 27 g at the start of the experiments. The mice were sacrificed when the tumors reached a diameter of 10 mm. For the liver metastasis model, live cells (1 × 10^6^ cells) were sorted by 7-AAD (Miltenyi Biotec) according to EGFP expression using a SH800 cell sorter (SONY) and injected into the spleen. Liver metastasis was assessed every 4 weeks. Mice were sacrificed 8 weeks after injection for the assessment of liver metastases in the POU5F1 expression metastatic ability experiment and 10 weeks after injection in the XAV939 experiment.

### Histological analysis

Xenograft tumors were fixed in formalin, processed through a series of graded concentrations of ethanol, embedded in paraffin, and sectioned. Sections were stained with hematoxylin and eosin (H&E). Three-dimensional (3D)-formed 2DOs cultured on a NanoCulture plate were collected and centrifuged at 400 × *g* for 5 min at room temperature. The pellet was consolidated using iPGell (GenoStaff Co., Ltd., Tokyo, Japan) and fixed in formalin. The pellet was processed through a series of graded concentrations of ethanol, embedded in paraffin, sectioned, and stained with H&E.

### Immunohistochemistry

Xenograft tumors were also fixed in 10% buffered formalin and embedded in paraffin blocks. For cultured 2DOs, 3D-formed 2DOs cultured on an Ultra-Low Attachment Multiple Well Plate (Corning, NY, USA) were collected and centrifuged at 400 × *g* for 5 min at room temperature. They were embedded in paraffin blocks using iPGell (GenoStaff). A 3-μm section was obtained from each block. Sections were deparaffinized, and slides were boiled for 15 min. Expressions of CD44, CK20, MUC2, and chromogranin A were quantified using antibodies (Supplementary Table S5). The slides were incubated with a primary antibody for 60 min at room temperature and then incubated with a secondary antibody for 30 min at room temperature. Slides were mounted in Prolong Gold with DAPI (Invitrogen, Waltham, MA, USA). Mucus production ability was assessed via Alcian blue staining (pH 2.5).

### Immunocytochemistry

Cultured cells were fixed with 4% formaldehyde and blocked. They were incubated with primary antibodies (Supplementary Table S6) overnight at 4 °C. Cells were incubated with secondary antibodies for 90 min. Slides were mounted in Prolong Gold with DAPI (ThermoFisher Scientific) overnight.

### Establishment of DsRed-Express2 expressing cells

The vector, pLV[Exp]-Neo-CMV>DsRed_Express2, was constructed by VectorBuilder, Inc. (Chicago, IL, USA) (Supplementary Fig. S27). This vector was transfected into 2DOs and iPS cells using Lentiviral High Titer Packaging Mix with pLVSIN (Takara Bio). DsRed_Express2-positive cells were selected by antibiotic selection using G418 (10131035; ThermoFisher Scientific) and sorted twice by an SH800 cell sorter (SONY). All cells expressing DsRed-Express2 were detected by an SH800 cell sorter (SONY).

### Establishment of POU5F1-EGFP cells with inducible caspase 9

The vector, PL-SIN-Oct4-EGFP, kindly provided by James Ellis (Addgene plasmid #21319)^22^, and the vector, pMSCV-F-del Casp9.IRES.GFP, kindly provided by David Spencer (Addgene plasmid # 15567)^46^, were used to establish cells expressing EGFP under the *OCT4 (POU5F1)* promoter with inducible caspase 9. Sequence-encoding caspase 9 was digested with restriction enzymes, *Xho*I (R0146S; New England Biolabs, Beverly, MA, USA) and *EcoR*I-HF (R3101S; New England Biolabs). The DNA fragment of caspase 9 was extracted from E-Gel CloneWel 0.8% (G6500ST; ThermoFisher Scientific) using the E-Gel Power Snap Electrophoresis System (ThermoFisher Scientific) (Supplementary Fig. S28). The fragment was amplified using CloneAmp HiFi PCR Premix (Z9298N; Takara Bio) with designed primers (FW_gaattctgcagtcgatcgagggagtgcaggtgg, RV_ccgcggtaccgtcgacttagtcgagtgcgtagtc). The vector, PL-SIN-Oct4-EGFP, was linearized by a restriction enzyme, *Sal*I-HF (R3138S; New England Biolabs). The amplified fragments and linearized vector were used for the cloning reaction by the In-Fusion HD Cloning Kit (Z9648N; Takara Bio). The transformation procedure was performed using Competent High *E. Coli* DH5α (TYB-DNA903; Toyobo, Osaka, Japan), and the plasmid was extracted using the Qiagen Plasmid Plus Midi Kit (12945; Qiagen). The nucleotide sequence of the vector was confirmed by Sanger sequencing, performed by GENEWIZ Japan Corp. (Kawaguchi, Japan). Primer extension sequencing was performed using Applied Biosystems BigDye version 3.1, and the reactions were then run on an Applied Biosystem 3730xl DNA Analyzer. The constructed vector was transfected into two 2DOs (603iCC and 25DiCC) using Lentiviral High Titer Packaging Mix with pLVSIN (Takara Bio). EGFP-positive cells were cloned by single-cell sorting using an SH800 cell sorter (SONY). *POU5F1* expression was confirmed by PCR, and a decrease in the number of EGFP-positive cells was confirmed by the administration of B/B Homodimerizer (Z5059N; Takara Bio). The mean provirus copy number was 6.05 (±1.16, *n* = 6), as measured using the Let-X Provirus Quantitation Kit (Z1239N; Takara Bio).

### Single-cell RNA sequencing and generation of the data matrix

603iCC-transfected POU5F1-EGFP cells with inducible caspase 9 (4.5 × 10^4^/well) were seeded, and 5 μM B/B Homodimerizer (Takara Bio) was administered for three days. Four days after the dimerizer was removed, live cells were sorted using an SH800 cell sorter (SONY) as “day 7 cells.” For cells not treated with a dimerizer, live cells were also sorted as “day 0 cells.” Single-cell library preparation was performed following the manufacturer’s instructions for the Chromium Next GEM Single Cell 3’ Reagent Kit (v3.1) (10x Genomics, Pleasanton, CA, USA), and the libraries were sequenced on a HiSeq X sequencer (Illumina). To generate a data matrix, the Cell Ranger pipeline (v4.0.0) was applied, and raw reads were aligned to the human reference genome (GRCh 38) using the STAR aligner. For GFP transcript mapping, the GFP sequence (XM_013393261) was added to the reference fastq and gtf files. Data were deposited in Gene Expression Omnibus under the accession number GSE169220.

### Data quality control and analysis

Seurat (version 3.2.0)^47^ was used for quality control and downstream analysis. Poor-quality cells were filtered out using the following parameters: nFeature_RNA 200–9000 and percent.mt <10. A total of 6942 cells (control: 3342 cells and day 7: 3602 cells), which passed the quality control, were finally used for further analysis. Mitochondrial genes were filtered by mt.percent (<10). UMAP visualization was used for dimensionality reduction analysis with the following parameters: resolution, 0.5; and perplexity, 20. Marker genes discriminating the different clusters were identified using the FindAllMarkers function (min.pct = 0.25 and log[fold-change] >0.25). Pathway enrichment analysis was performed using Enrichr^48^ (https://maayanlab.cloud/Enrichr/). To construct a single-cell pseudotime trajectory, the Monocle3 (v0.2.2) algorithm was applied (https://cole-trapnell-lab.github.io/monocle3/). After converting the Seurat object using the *as.cell_data_set* function, the root node was assigned to cluster 4, and the *orderCells* function was used to assign cells a pseudotime value. To subdivide cells based on their branch in the trajectory, the *choose_graph_segments* function was applied, and cluster 6 was chosen as an ending node.

### Western blot analysis

Western blot analysis was performed to examine proteins associated with the Wnt/β-catenin signaling pathway. Cells were lysed in 50 mM Tris–HCl (pH 7.6), 1% Nonidet P-40, 150 mM sodium chloride, and 0.1 mM zinc acetate in the presence of protease inhibitors. Protein concentration was determined by the Lowry method (Bio-Rad), and 20 µg of each sample was separated by 10% sodium dodecyl sulfate polyacrylamide gel electrophoresis. The gel was transferred electrophoretically onto a polyvinylidene difluoride membrane (Millipore, Billerica, MA, USA). The membrane was blocked with blocking buffer for 1 h and then incubated overnight at 4 °C with primary antibodies against β-catenin (1:1000, 8480, Cell Signaling Technology), Wnt-3a (1:5000, GTX128101, Gene Tex, CA, USA), and HistoneH3 (1:2000, 4499 S, Cell Signaling Technology). After a 2-h incubation with the secondary antibody, horseradish peroxidase-conjugated rabbit antibody (1:400, 7074 S, Santa Cruz Biotechnology Inc., Dallas, TX, USA), protein bands were visualized using an ECL detection kit (ThermoFisher Scientific) according to the manufacturer’s instructions.

### Methylation analysis

DNA samples were treated with sodium bisulfite using a bisulfite conversion kit (Zymo Research EZ DNA methylation Kit). After treatment, unmethylated cytosines convert to uracil, while methylated cytosines remain unchanged. Bisulfite-converted DNA samples were analyzed using the Infinium MethylationEPIC BeadChip Kit (Illumina). Bisulfite-converted DNA samples were denatured and neutralized by alkali. The denatured samples were then amplified by whole-genome amplification (37 °C overnight). Amplified DNA samples were enzymatically fragmented for 1 h at 37 °C in a microsample incubator. 2-Propanol was added to the fragmented DNA samples and precipitated by centrifugation. Precipitated DNA samples were resuspended with hybridization buffer and incubated for 1 h at 48 °C in a hybridization oven. Fragmented and resuspended DNA samples were denatured for 20 min at 95 °C in a microsample incubator. Denatured DNA samples were dispensed onto BeadChips using a TECAN System. The BeadChips were incubated overnight at 48 °C in the hybridization oven to hybridize the samples onto the BeadChips. After hybridization, seals were removed from the hybridized BeadChips. Next, unhybridized fragment DNAs were washed away. Labeled nucleotides were added to the washed BeadChips to extend primers which hybridized to the DNA. BeadChips were stained, then coated for protection, and dried. Dried BeadChips were scanned with the iSCAN System. Illumina GenomeStudio software (V2011.1) loaded the signal intensity files of BeadChips, and beta values were decided via normalization and background subtraction. Next, a comparative analysis was executed based on the Illumina Custom Model algorithm, and difference scores for all probes were computed. The markers with signal intensities adequate to distinguish between the signal and background noise were used in subsequent analysis. The markers with high scores (highly methylated and highly unmethylated compared to the reference sample) were extracted, and clustering analysis was conducted.

### Analysis of the binding energy

The NANOG binding consensus sequence is generally known to be 5′–TAAT[GT][GT]–3′ or 5′–[CG][GA][CG]C[GC]ATTAN[GC]–3′. Therefore, in the sequence of focus, the CGCCCAGTGTC part is quite similar to the binding sequence. We used Protein Data Bank data, including 4RBO, to predict binding conformations to the NANOG protein with the wild-type sequences or methylated sequence with our original method^49^. A sufficient amount of water molecules was placed around the complex structures, and thermodynamical sampling was performed under a periodic boundary condition. After stabilizing the complex structure by energy minimization calculations, some molecular dynamics simulations were performed at ~37 °C (310 °K) to capture the molecular behavior under the biological environment. After a sufficient thermal equilibration process, the molecular vibrations of the bonding configurations were sampled. All these calculations were performed using the AMBER package. The distributions of the interaction energy between DNA and NANOG protein were calculated by extracting 2000 conformations of complex structures from the trajectory with the abovementioned molecular dynamics simulations. Each binding energy was calculated using Gaussian program packages^50^ with the AMBER99 Force field level^51^.

### In vitro proliferation assays for a Wnt inhibitor

603iCC cells (1 × 10^4^ per well) were seeded into 96-well plates and incubated for 48 h. After incubation, cells were exposed to different concentrations of XAV939 (BD248591; BLD Phamatech Ltd., Shanghao, China) for 96 h. The percentage of viable cells was determined using a cell counting kit solution (CCK-8; Dojindo Molecular Technologies) according to the manufacturer’s protocol.

### Analysis of floating cancer cell counts with a Wnt inhibitor

Prior to cancer cell seeding, plates were coated. iPS cell-coated plates were seeded into 12-well plates (2 × 10^5^ iPS cells/well) 2 days prior to seeding. iPS cells were tagged with DsRed-Express by the aforementioned methods. Laminin coatings were prepared using iMatrix-511 (T304, Takara Bio) according to the manufacturer’s protocol. Sorted POU5F1-positive cells (2 × 10^5^/ well) were seeded on these plates. Medium was prepared with XAV939 (10 μM) for the XAV939 group and DMSO (0.3%) for the control group. All medium exchanges were performed every other day, and cells in the collected supernatant were analyzed by an SH800 cell sorter (SONY). Cells not expressing DsRED-Express2 were counted as cancer cells.

### Expression analysis of Flower on immunocytochemistry

Stained specimens were analyzed using ImageJ software^52^. Five independent images were collected for each sample and the areas of protein expression in the samples were measured. The value was normalized by dividing by the number of cells stained with DAPI.

### Evaluation of XAV939 using a liver metastasis model of CRC

As an evaluation of XAV939, sorted POU5F1-positive cells were directly injected into the spleen of mice (1 × 10^6^ cells). After recovering from anesthesia, mice were randomly allocated to the control (0.3% DMSO that is the final concentration of DMSO in XAV939 group) or XAV939 group (100 μg/injection/mouse). XAV939 (CS-0494, ChemScene, Monmouth Junction, NJ, USA) was administered by intraperitoneal injection at 1 mg/mL (injection volume, 100 µL) every day for 8 weeks, followed by 2 weeks of observation. Ten weeks after injection, mice were sacrificed for the assessment of metastases. Mouse body weight was measured twice per week, and no weight gain or loss greater than 5% was observed.

### Ethics approval and consent to participate

The Osaka University Review Board, the OICI Review Board, approved this study, and written informed consent for the study was obtained from all participants according to the ethics guidelines. All ethical regulations relevant to human research participants were followed. The OICI Animal Research Committee approved this study, and we have complied with all relevant ethical regulations for animal use. All experimental protocols were in accordance with the guidelines of the Osaka University, the OICI, and Declaration of Helsinki.

### Statistics and reproducibility

Continuous variables are expressed as the mean with standard error of the mean. The significance of the difference between the two groups was analyzed using the *x*^2^ test and Wilcoxon’s signed rank-sum test. All data were analyzed using JMP software (SAS Institute), R 3.6.3, and Prism 8 (GraphPad Software, San Diego, CA, USA). Results were considered statistically significant at *P* < 0.05.

### Supplementary information

Supplementary Information

Description of additional supplementary files

Supplementary Data 1

Supplementary Data 2

Supplementary Movie 1

## Data Availability

The scRNA sequence datasets of this study are available in the Gene Expression Omnibus under the accession number GSE169220. Data that were presented in figures were deposited in figsue (10.6084/m9.figshare.24447370).
