# Supplementary material for: Metastases and treatment-resistant lineages in patient-derived cancer cells of colorectal cancer
Source: Commun Biol. 2023 Nov 24;6:1191. doi: 10.1038/s42003-023-05562-y (PMC10667365; doi:10.1038/s42003-023-05562-y)
Supplement: Supplementary file 1 — Supplementary Information [file 42003_2023_5562_MOESM1_ESM.docx]

**Supplemental Figure S1**


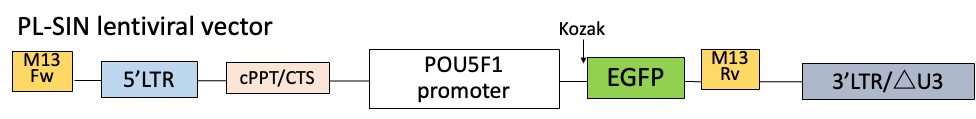


Overview of the POU5F1-EGFP expressing vector

**Supplemental Figure S2**

POU5F1 expression in EGFP-negative and EGFP-positive cells of DsRed-Express2-positive cells by qRT-PCR (*n=*5, ***P*<0.01, mean value ± SEM)

**Supplemental Figure S3**


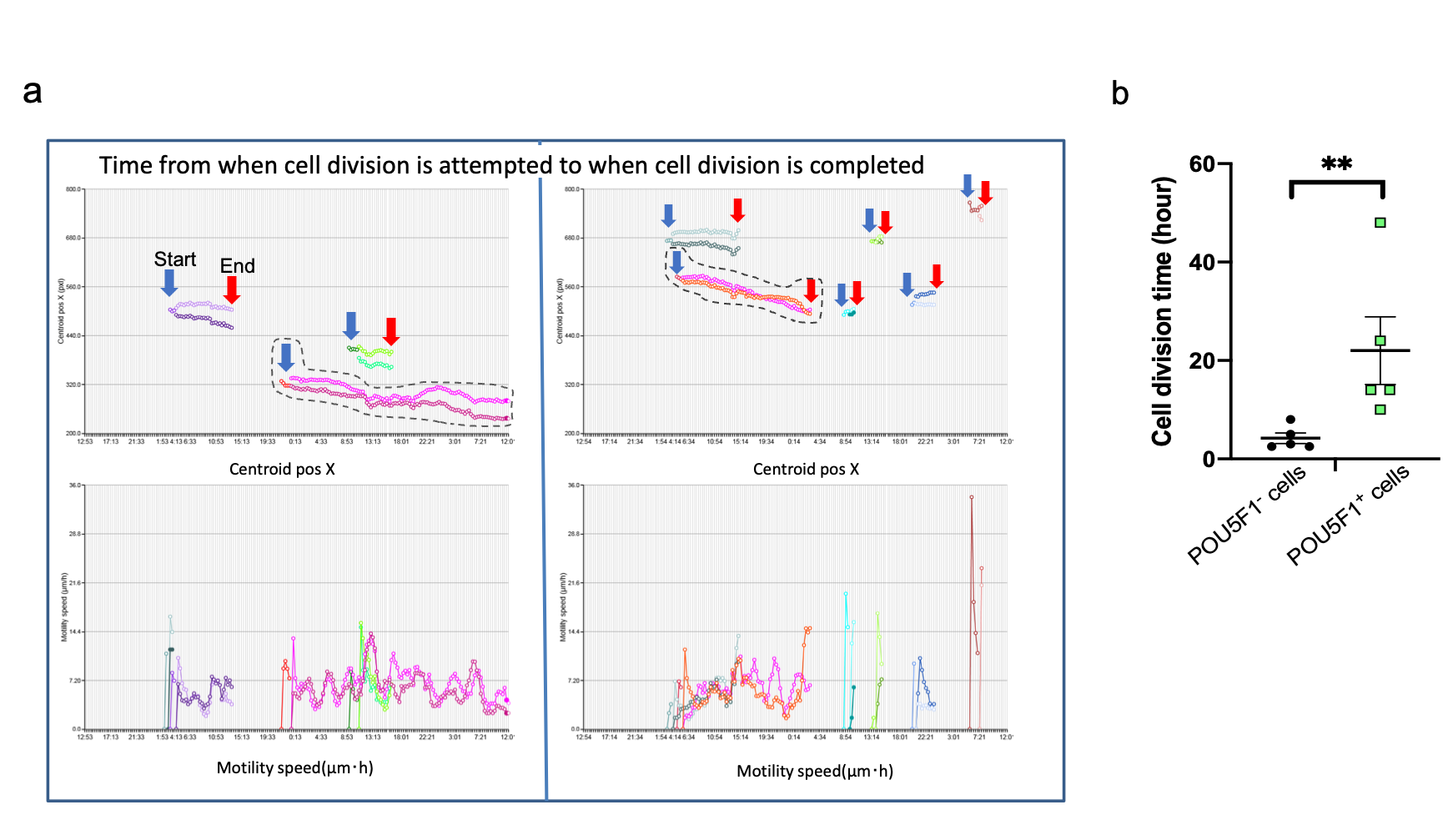


(a) Examination of cell division time by measurement of cell movement using live cell imaging. Long cell divisions surrounded by dotted lines were only observed in EGFP-positive cells. (b) Cell division time of POU5F1-positive/negative cells. (n=5, ***P*<0.01, mean value ± SEM)

**Supplemental Figure S4**

**
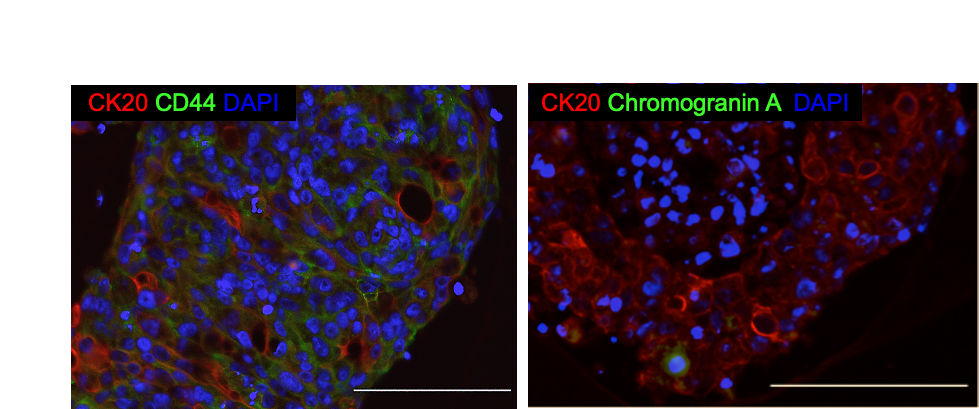
**

Tissue immunostaining of cells derived from a single EGFP-positive cell. CK20, CD44 and Chromogranin A in *in vitro* population of 2DOs (603iCC). (Scale bars, 100μm)

**Supplemental Figure S5**

**
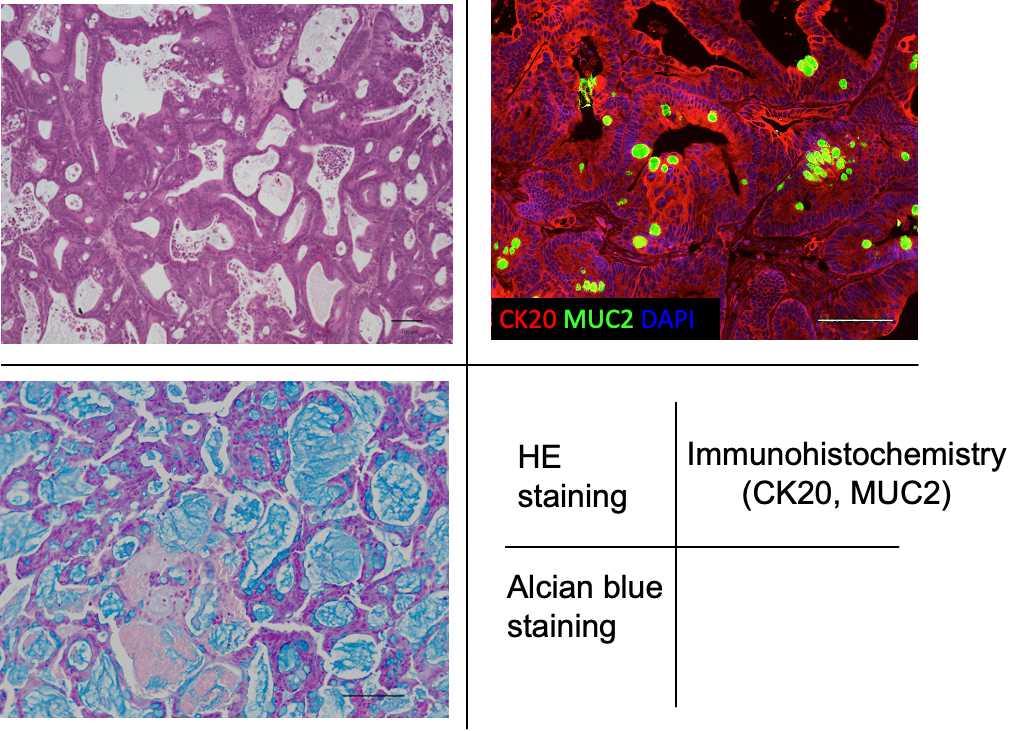
**

Tissue immunostaining with Alcian blue and MUC2 for evaluating *in vivo* tumor by 2DOs (603iCC). (Scale bars, 100μm)

**Supplemental Figure S6**

**
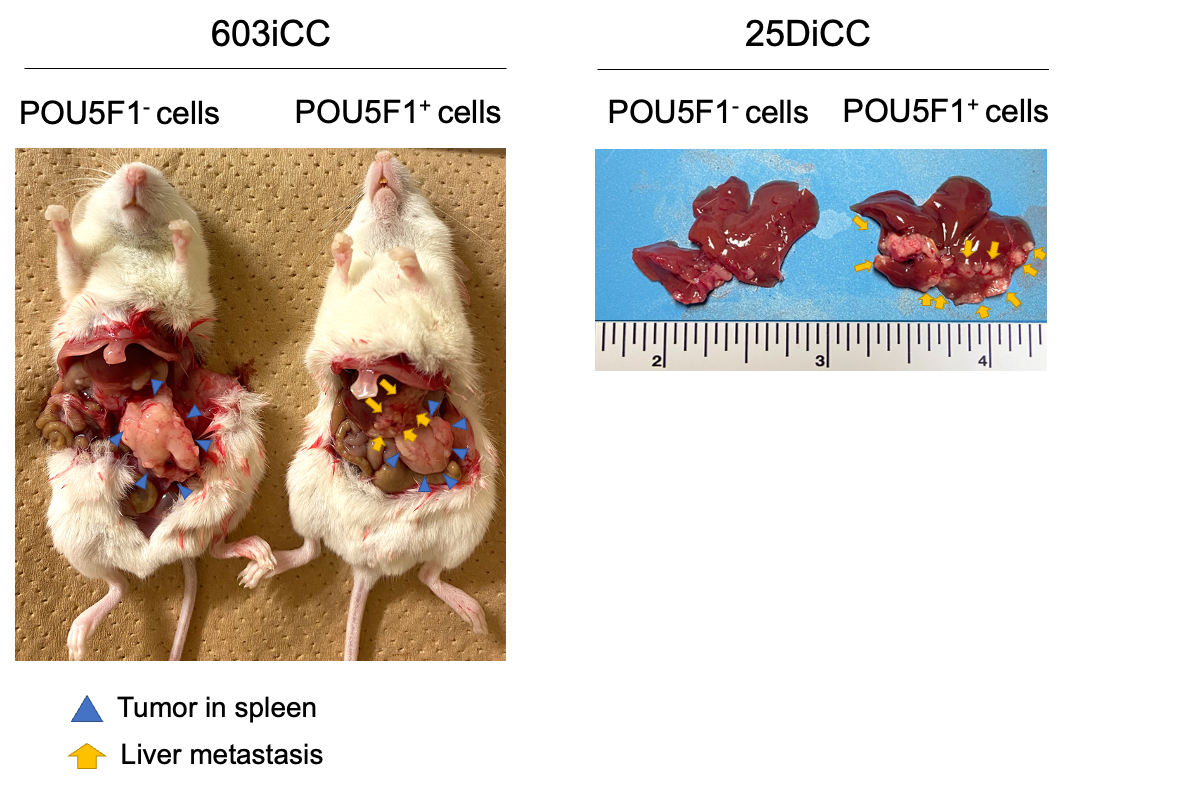
**

Representative macroscopic image of the liver upon intra-splenic administration of POU5F1-positive and POU5F1-negative cells in two 2DOs (603iCC and 25DiCC).

**Supplemental Figure S7**

POU5F1 and CASP9 expression in dimerizer treated cells by qRT-PCR (*n=*3, **P*<0.05, mean value ± SEM)

**Supplemental Figure S8**

**
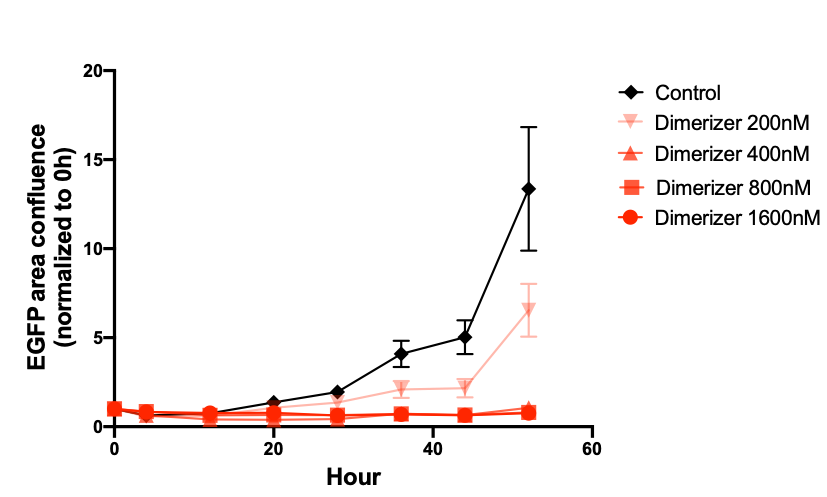
**

EGFP-positive cell counts upon administration of increasing doses of the dimerizer (*n=*4, median value ± SEM).

**Supplemental Figure S9**

**
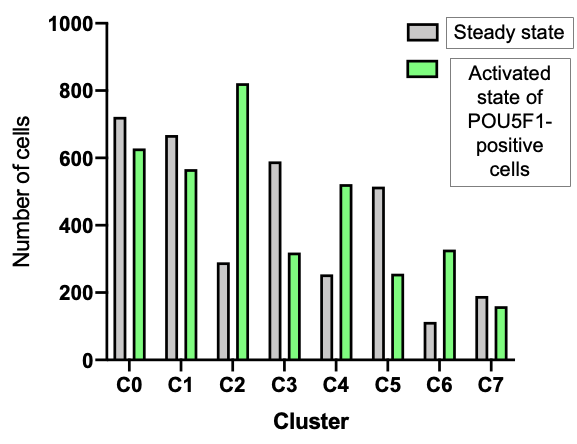
**

Number of cells classified in each cluster.

**Supplemental Figure S10**

**
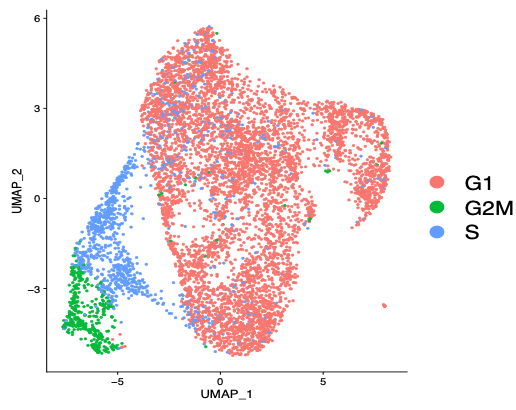
**

Cell cycle score analysis

**Supplemental Figure S11**

**
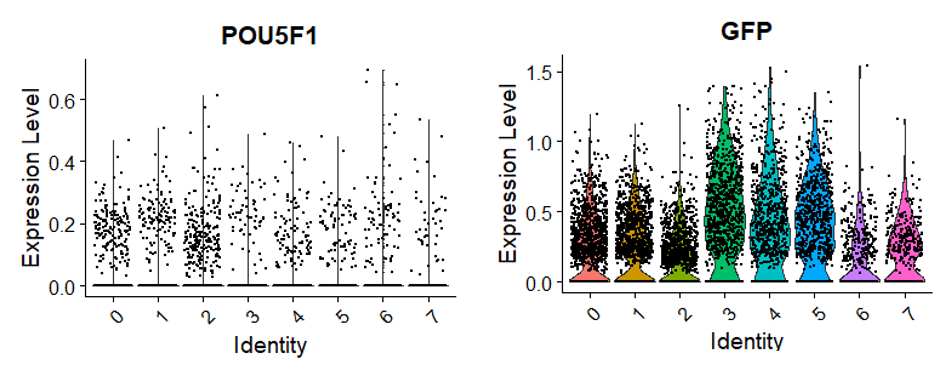
**

Violin plots of POU5F1 and EGFP expression in each clusters.

**Supplemental Figure S12**

**
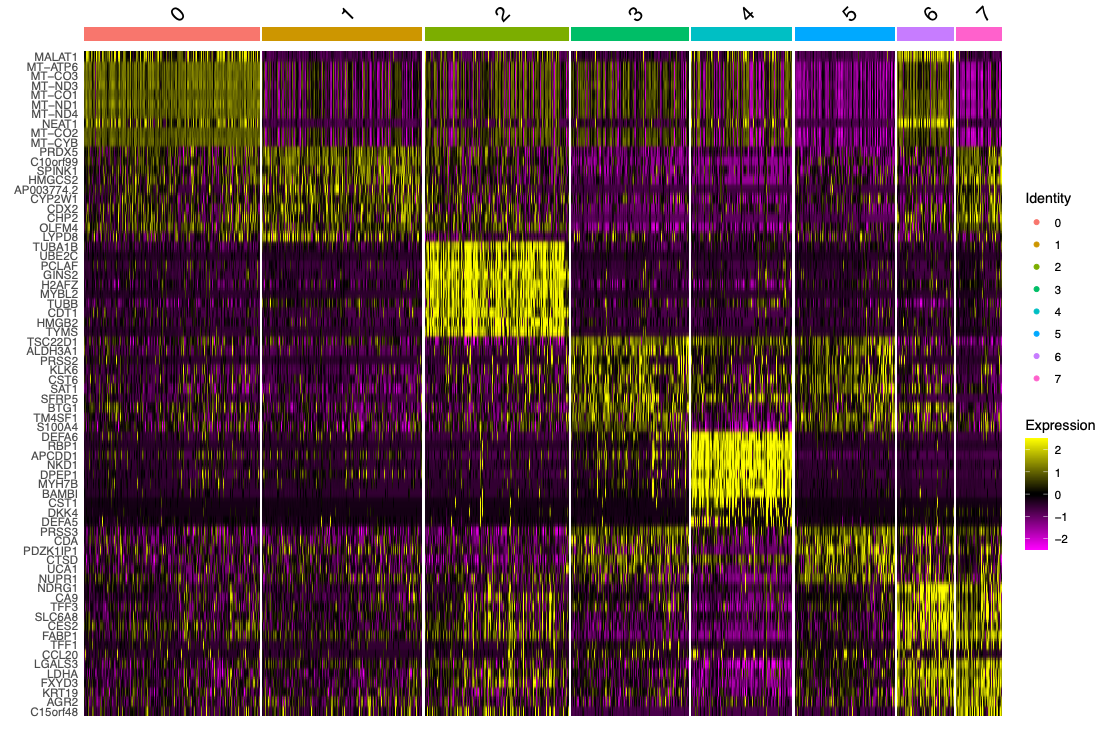
**

Representative genes characteristic of each cluster.

**Supplemental Figure S13**

**
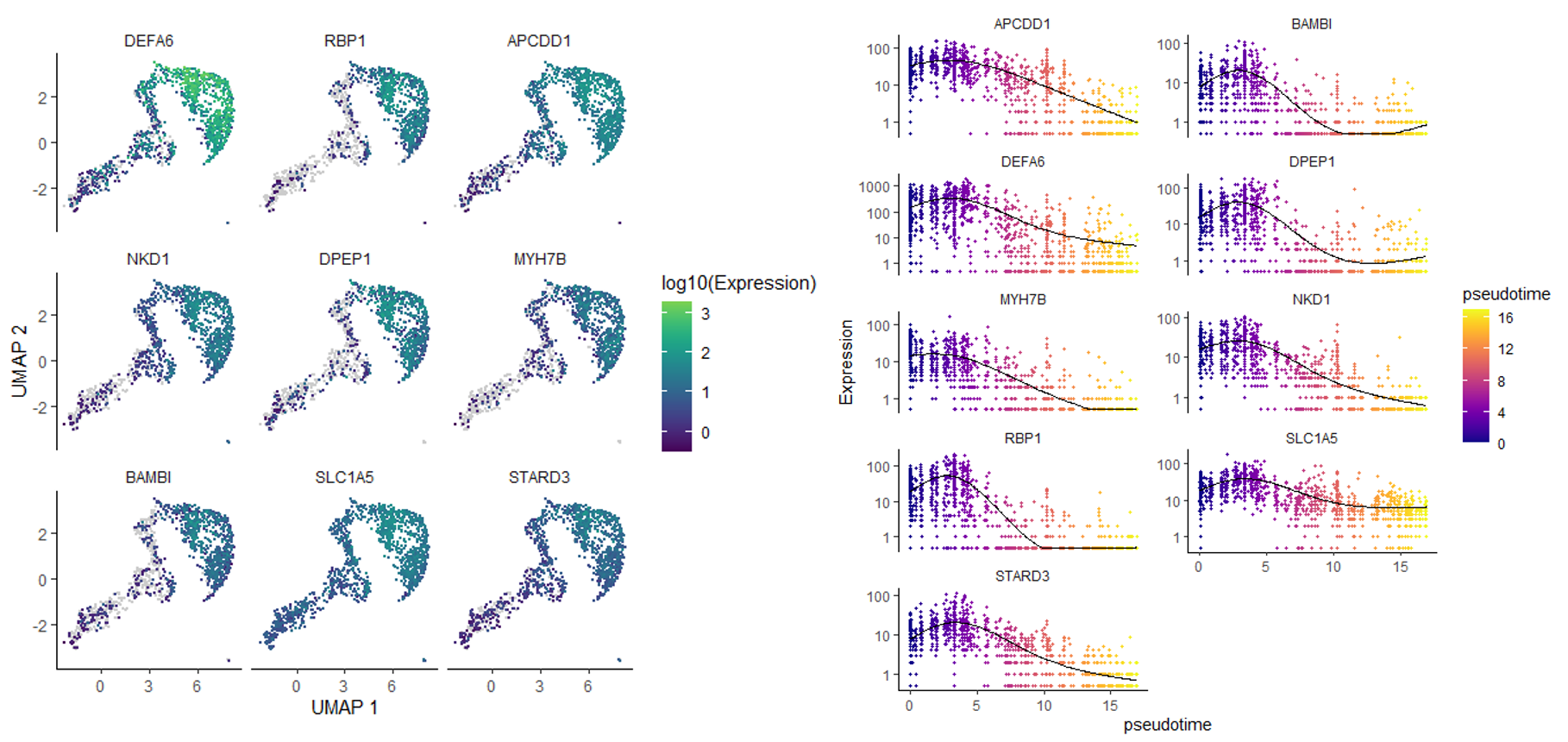
**

Typical genes whose expression decreases as the pseudo time progresses.

**Supplemental Figure S14**

**
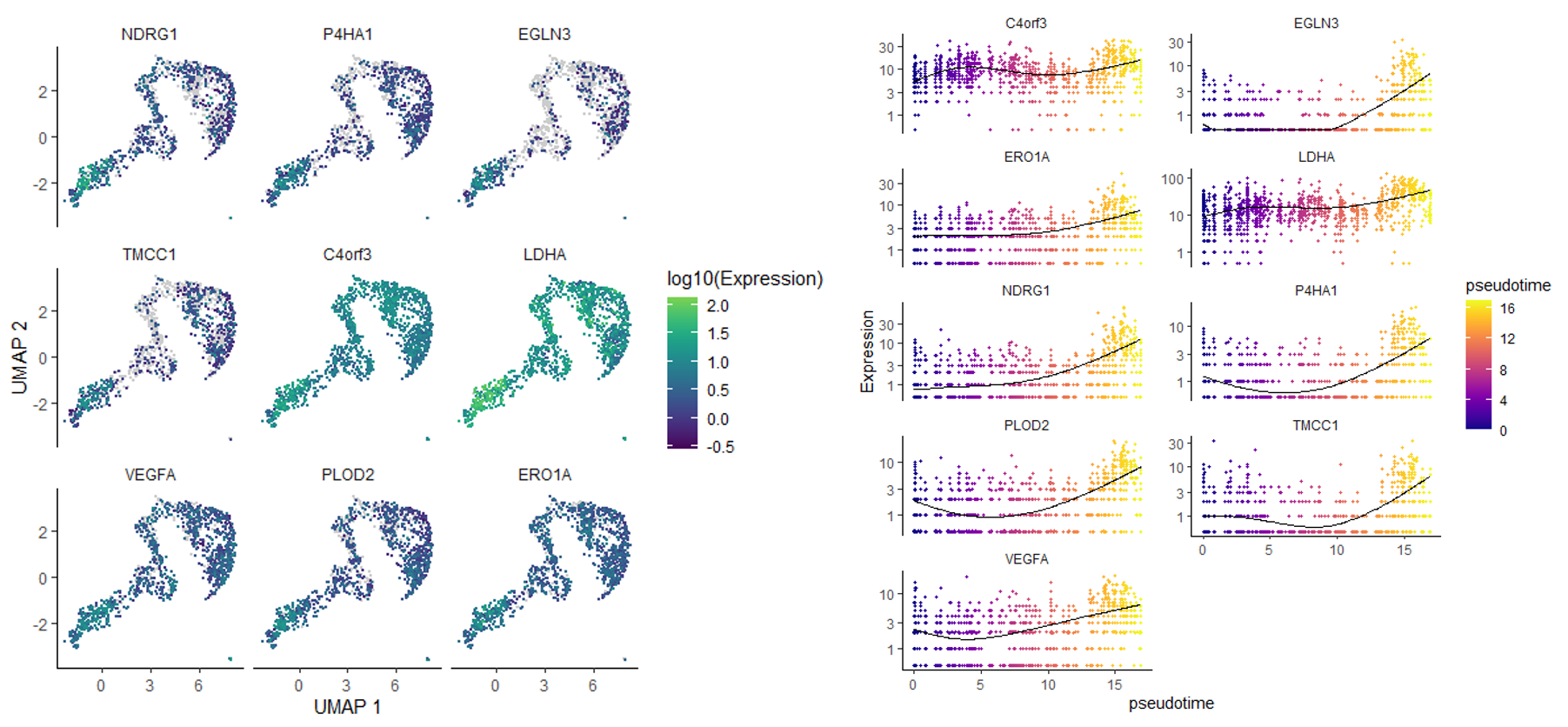
**

Typical genes whose expression increases as the pseudo time progresses.

**Supplemental Figure S15**

**
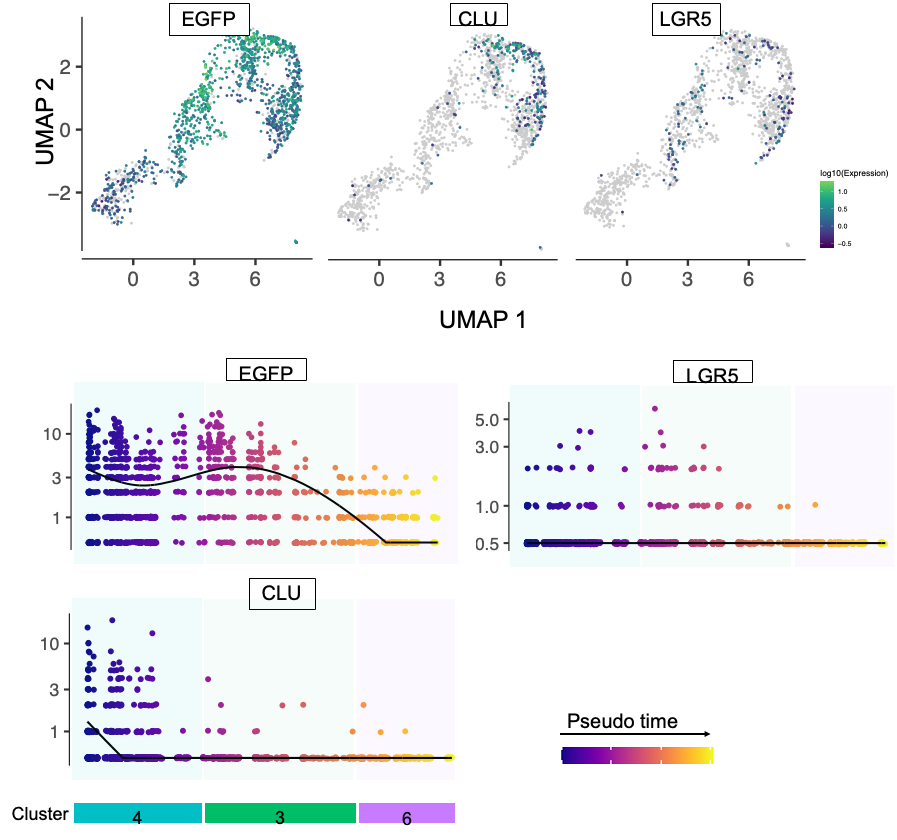
**

Expression of EGFP, CLU, and LGR5 in clusters 4, 3, and 6, according to pseudo-time.

**Supplemental Figure S16**

**
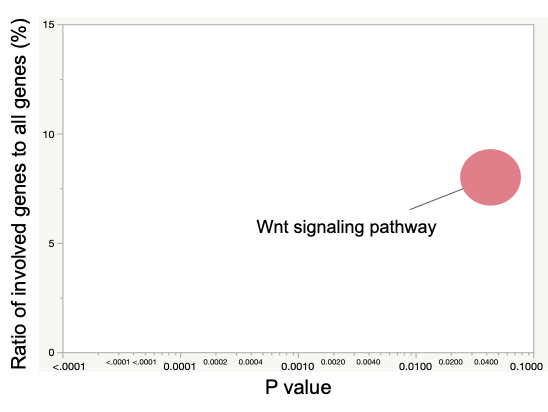
**

Enriched pathways in cluster 4.

**Supplemental Figure S17**

Wnt-3a expression in EGFP-negative cells, EGFP-positive cells, cells treated with 0.3% DMSO (as control) for 96 h, and cells treated with 500 nM dimerizer for 96 h by western blotting. (603iCC, *n=*3, **P*<0.05, mean value ± SEM)

**Supplemental Figure S18**

**
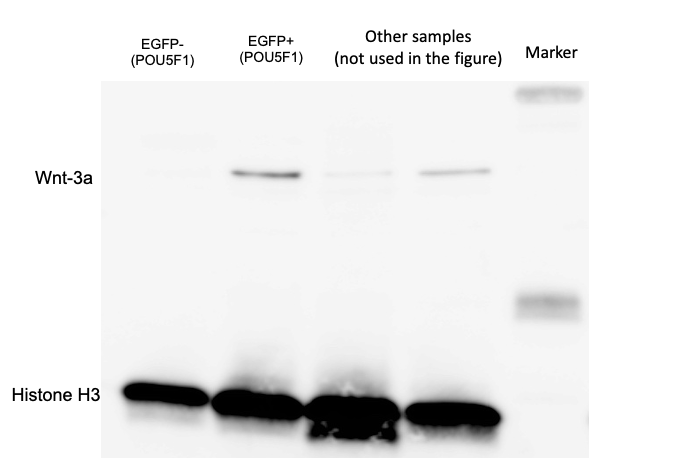
** **
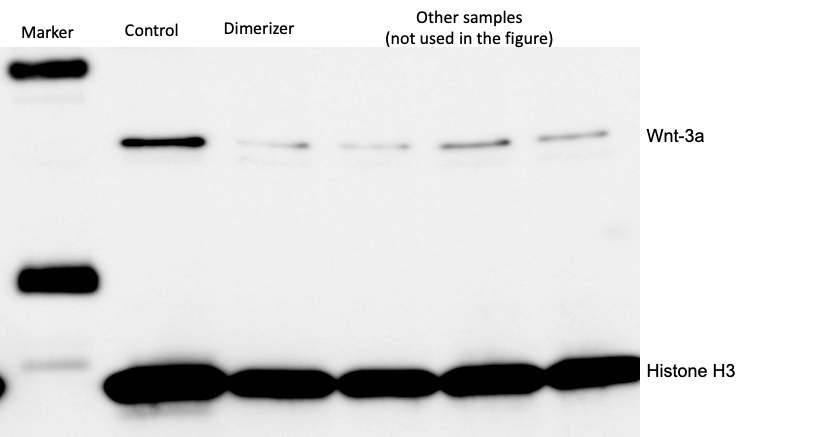
**

Uncropped gel image for Figure 5j.

**Supplemental Figure S19**

**
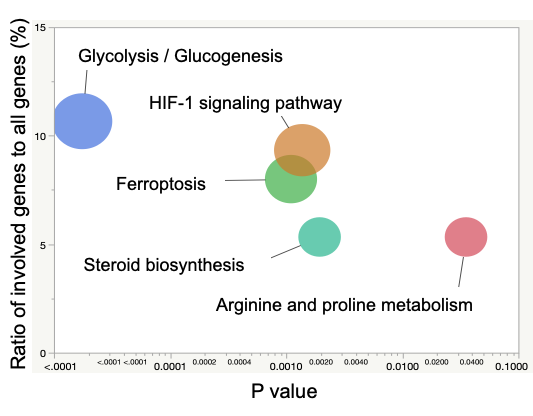
**

Enriched pathways in cluster 6.

**Supplemental Figure S20**

**
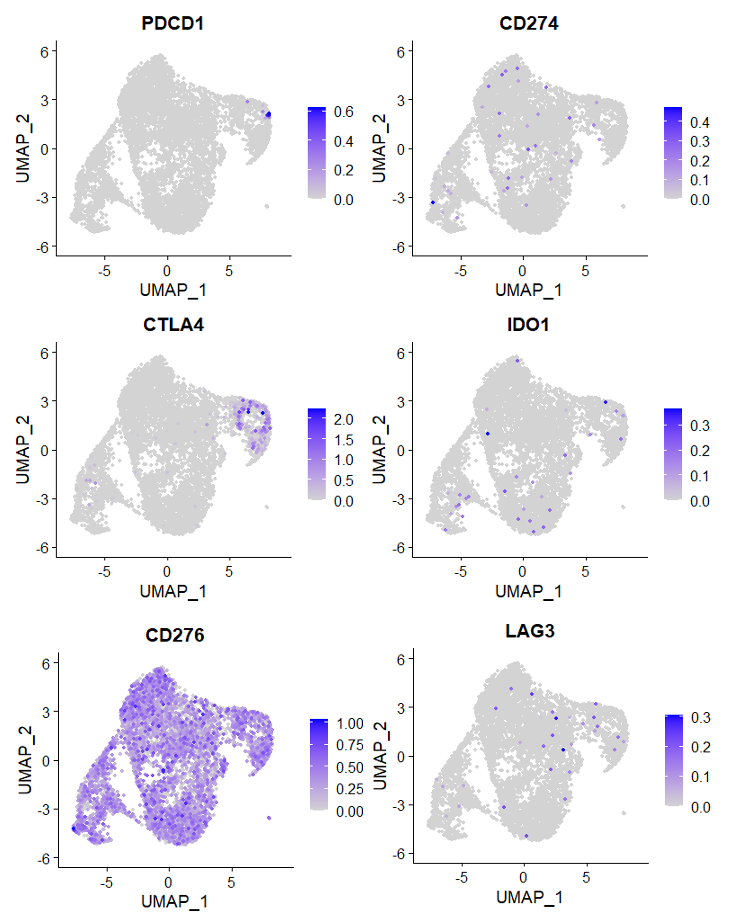
**

Expression of immune-related genes

**Supplemental Figure S21**

**
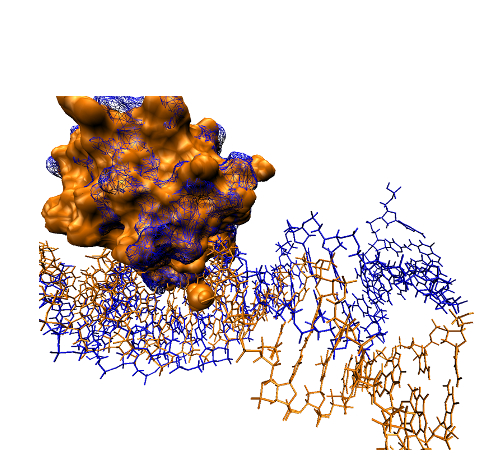
**

Representative superposition of binding conformations of WT sequences to and methylated sequences to NANOG. The blue and orange one means the binding conformation of WT and of methylated form, respectively. In this figure, the two binding conformations are superimposed so that the Backbone of the NANOG protein overlaps the most.

**Supplemental Figure S22**

β-Catenin expression in EGFP-negative cells, EGFP-positive cells, cells treated with 0.3% DMSO (as control) for 96 h, cells treated with 500 nM dimerizer for 96 h, and cells treated with 10μM XAV939 for 96 h by western blotting. (603iCC, *n=*3, **P*<0.05, mean value ± SEM)

**Supplemental Figure S23**

**
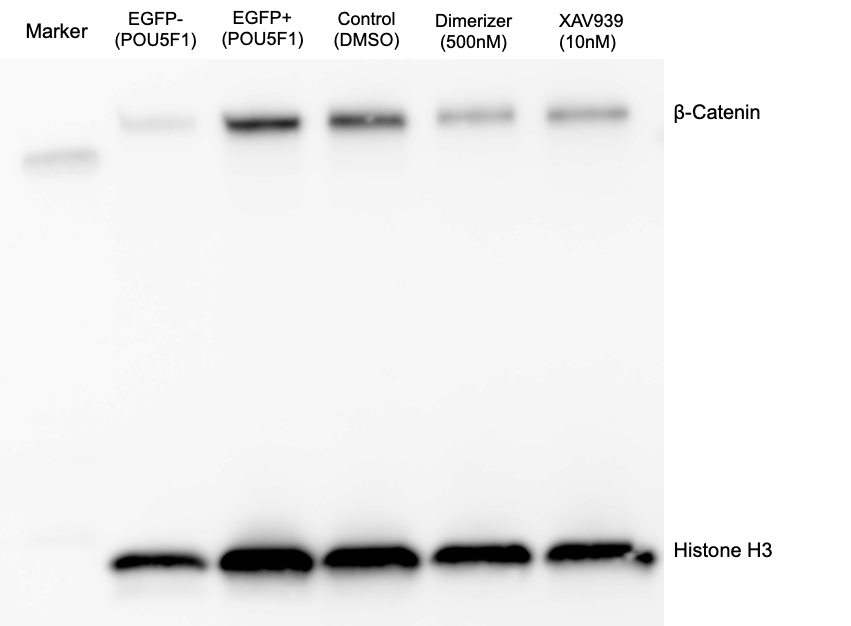
**

Uncropped gel image for Figure 6a.

**Supplemental Figure S24**

Wnt-3a expression in cells treated with DMSO (as control) for 96 h, and cells treated with 10μM XAV939 for 96 h by western blotting. (603iCC, *n=*3, **P*<0.05, mean value ± SEM)

**Supplemental Figure S25**

**
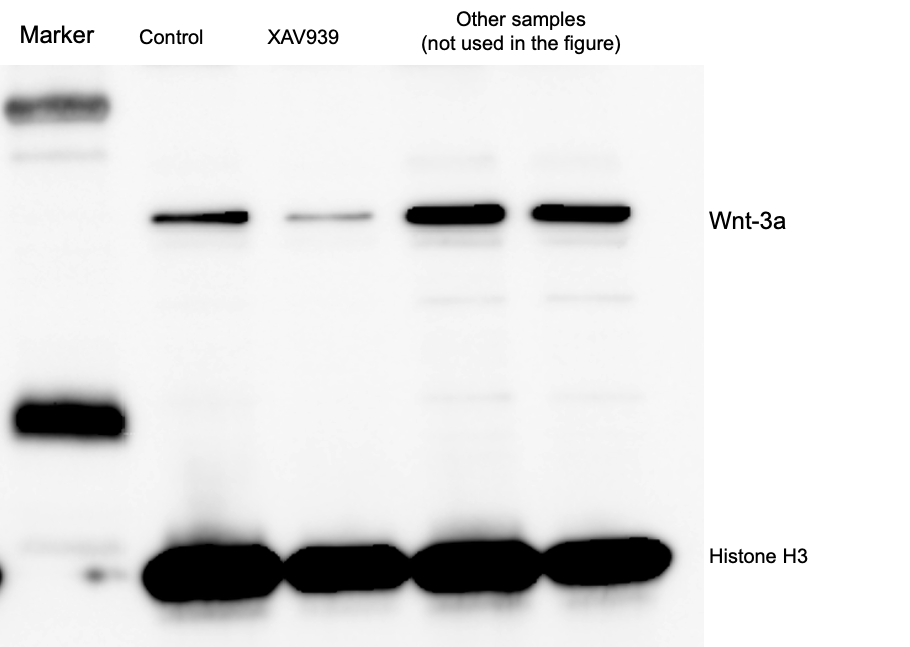
**

Uncropped gel image for Figure 6b.

**Supplemental Figure S26**

**
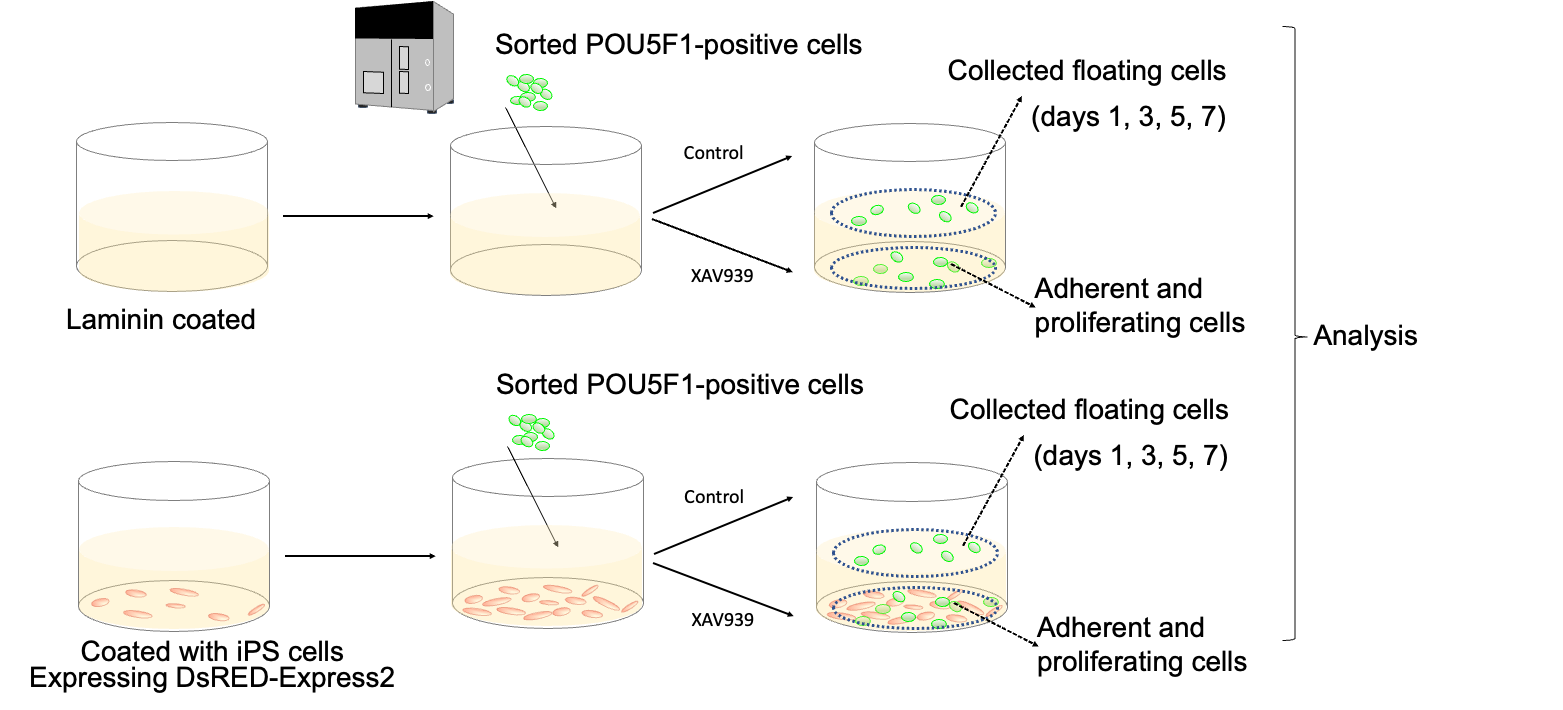
**

Schema of analysis evaluating the effects of Wnt inhibitor (XAV939) on adhesion and proliferation of sorted POU5F1-positive cells.

**Supplemental Figure S27**

**Vector map of pLV[Exp]-Neo-CMV>DsRed_Express2.**


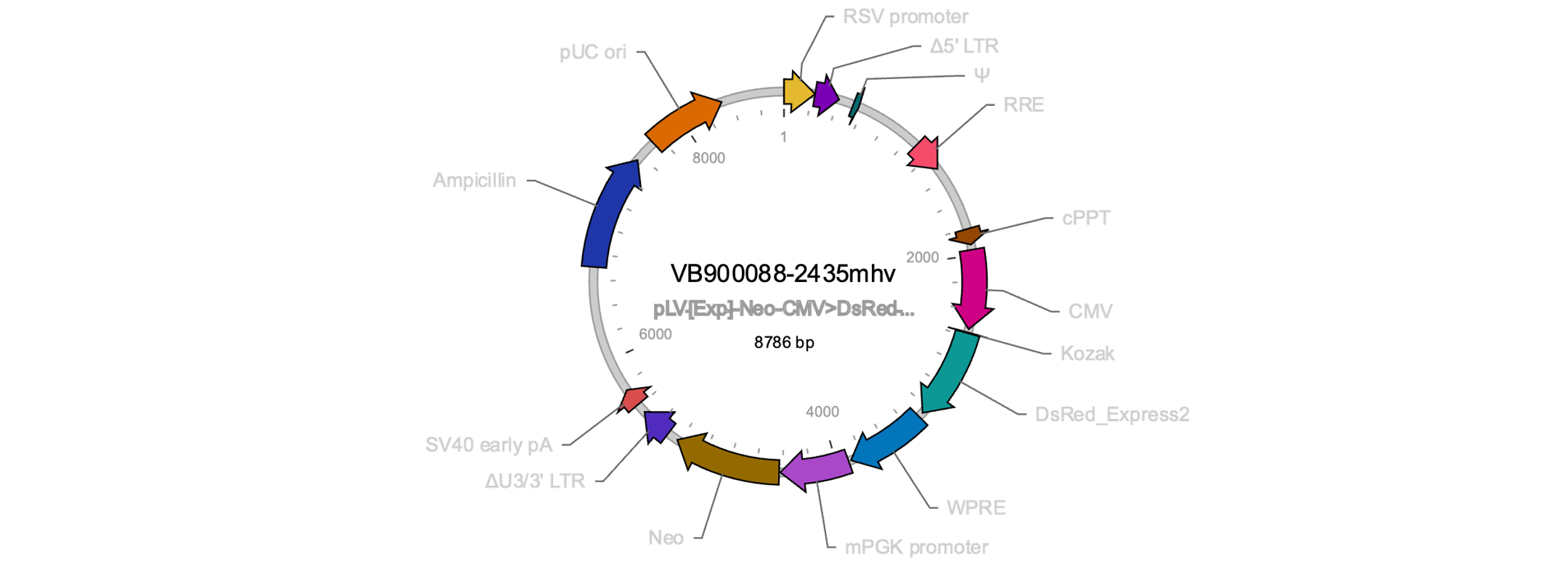


**Vector sequence**

AATGTAGTCTTATGCAATACTCTTGTAGTCTTGCAACATGGTAACGATGAGTTAGCAACA

TGCCTTACAAGGAGAGAAAAAGCACCGTGCATGCCGATTGGTGGAAGTAAGGTGGTACGA

TCGTGCCTTATTAGGAAGGCAACAGACGGGTCTGACATGGATTGGACGAACCACTGAATT

GCCGCATTGCAGAGATATTGTATTTAAGTGCCTAGCTCGATACATAAACGGGTCTCTCTG

GTTAGACCAGATCTGAGCCTGGGAGCTCTCTGGCTAACTAGGGAACCCACTGCTTAAGCC

TCAATAAAGCTTGCCTTGAGTGCTTCAAGTAGTGTGTGCCCGTCTGTTGTGTGACTCTGG

TAACTAGAGATCCCTCAGACCCTTTTAGTCAGTGTGGAAAATCTCTAGCAGTGGCGCCCG

AACAGGGACTTGAAAGCGAAAGGGAAACCAGAGGAGCTCTCTCGACGCAGGACTCGGCTT

GCTGAAGCGCGCACGGCAAGAGGCGAGGGGCGGCGACTGGTGAGTACGCCAAAAATTTTG

ACTAGCGGAGGCTAGAAGGAGAGAGATGGGTGCGAGAGCGTCAGTATTAAGCGGGGGAGA

ATTAGATCGCGATGGGAAAAAATTCGGTTAAGGCCAGGGGGAAAGAAAAAATATAAATTA

AAACATATAGTATGGGCAAGCAGGGAGCTAGAACGATTCGCAGTTAATCCTGGCCTGTTA

GAAACATCAGAAGGCTGTAGACAAATACTGGGACAGCTACAACCATCCCTTCAGACAGGA

TCAGAAGAACTTAGATCATTATATAATACAGTAGCAACCCTCTATTGTGTGCATCAAAGG

ATAGAGATAAAAGACACCAAGGAAGCTTTAGACAAGATAGAGGAAGAGCAAAACAAAAGT

AAGACCACCGCACAGCAAGCGGCCGCTGATCTTCAGACCTGGAGGAGGAGATATGAGGGA

CAATTGGAGAAGTGAATTATATAAATATAAAGTAGTAAAAATTGAACCATTAGGAGTAGC

ACCCACCAAGGCAAAGAGAAGAGTGGTGCAGAGAGAAAAAAGAGCAGTGGGAATAGGAGC

TTTGTTCCTTGGGTTCTTGGGAGCAGCAGGAAGCACTATGGGCGCAGCGTCAATGACGCT

GACGGTACAGGCCAGACAATTATTGTCTGGTATAGTGCAGCAGCAGAACAATTTGCTGAG

GGCTATTGAGGCGCAACAGCATCTGTTGCAACTCACAGTCTGGGGCATCAAGCAGCTCCA

GGCAAGAATCCTGGCTGTGGAAAGATACCTAAAGGATCAACAGCTCCTGGGGATTTGGGG

TTGCTCTGGAAAACTCATTTGCACCACTGCTGTGCCTTGGAATGCTAGTTGGAGTAATAA

ATCTCTGGAACAGATTTGGAATCACACGACCTGGATGGAGTGGGACAGAGAAATTAACAA

TTACACAAGCTTAATACACTCCTTAATTGAAGAATCGCAAAACCAGCAAGAAAAGAATGA

ACAAGAATTATTGGAATTAGATAAATGGGCAAGTTTGTGGAATTGGTTTAACATAACAAA

TTGGCTGTGGTATATAAAATTATTCATAATGATAGTAGGAGGCTTGGTAGGTTTAAGAAT

AGTTTTTGCTGTACTTTCTATAGTGAATAGAGTTAGGCAGGGATATTCACCATTATCGTT

TCAGACCCACCTCCCAACCCCGAGGGGACCCGACAGGCCCGAAGGAATAGAAGAAGAAGG

TGGAGAGAGAGACAGAGACAGATCCATTCGATTAGTGAACGGATCTCGACGGTATCGCTA

GCTTTTAAAAGAAAAGGGGGGATTGGGGGGTACAGTGCAGGGGAAAGAATAGTAGACATA

ATAGCAACAGACATACAAACTAAAGAATTACAAAAACAAATTACAAAAATTCAAAATTTT

ACTAGTGATTATCGGATCAACTTTGTATAGAAAAGTTGTAGTTATTAATAGTAATCAATT

ACGGGGTCATTAGTTCATAGCCCATATATGGAGTTCCGCGTTACATAACTTACGGTAAAT

GGCCCGCCTGGCTGACCGCCCAACGACCCCCGCCCATTGACGTCAATAATGACGTATGTT

CCCATAGTAACGCCAATAGGGACTTTCCATTGACGTCAATGGGTGGAGTATTTACGGTAA

ACTGCCCACTTGGCAGTACATCAAGTGTATCATATGCCAAGTACGCCCCCTATTGACGTC

AATGACGGTAAATGGCCCGCCTGGCATTATGCCCAGTACATGACCTTATGGGACTTTCCT

ACTTGGCAGTACATCTACGTATTAGTCATCGCTATTACCATGGTGATGCGGTTTTGGCAG

TACATCAATGGGCGTGGATAGCGGTTTGACTCACGGGGATTTCCAAGTCTCCACCCCATT

GACGTCAATGGGAGTTTGTTTTGGCACCAAAATCAACGGGACTTTCCAAAATGTCGTAAC

AACTCCGCCCCATTGACGCAAATGGGCGGTAGGCGTGTACGGTGGGAGGTCTATATAAGC

AGAGCTGGTTTAGTGAACCGTCAGATCCAAGTTTGTACAAAAAAGCAGGCTGCCACCATG

GATAGCACTGAGAACGTCATCAAGCCCTTCATGCGCTTCAAGGTGCACATGGAGGGCTCC

GTGAACGGCCACGAGTTCGAGATCGAGGGCGAGGGCGAGGGCAAGCCCTACGAGGGCACC

CAGACCGCCAAGCTGCAGGTGACCAAGGGCGGCCCCCTGCCCTTCGCCTGGGACATCCTG

TCCCCCCAGTTCCAGTACGGCTCCAAGGTGTACGTGAAGCACCCCGCCGACATCCCCGAC

TACAAGAAGCTGTCCTTCCCCGAGGGCTTCAAGTGGGAGCGCGTGATGAACTTCGAGGAC

GGCGGCGTGGTGACCGTGACCCAGGACTCCTCCCTGCAGGACGGCACCTTCATCTACCAC

GTGAAGTTCATCGGCGTGAACTTCCCCTCCGACGGCCCCGTAATGCAGAAGAAGACTCTG

GGCTGGGAGCCCTCCACCGAGCGCCTGTACCCCCGCGACGGCGTGCTGAAGGGCGAGATC

CACAAGGCGCTGAAGCTGAAGGGCGGCGGCCACTACCTGGTGGAGTTCAAGTCAATCTAC

ATGGCCAAGAAGCCCGTGAAGCTGCCCGGCTACTACTACGTGGACTCCAAGCTGGACATC

ACCTCCCACAACGAGGACTACACCGTGGTGGAGCAGTACGAGCGCGCCGAGGCCCGCCAC

CACCTGTTCCAGTAGACCCAGCTTTCTTGTACAAAGTGGTGATAATCGAATTCCGATAAT

CAACCTCTGGATTACAAAATTTGTGAAAGATTGACTGGTATTCTTAACTATGTTGCTCCT

TTTACGCTATGTGGATACGCTGCTTTAATGCCTTTGTATCATGCTATTGCTTCCCGTATG

GCTTTCATTTTCTCCTCCTTGTATAAATCCTGGTTGCTGTCTCTTTATGAGGAGTTGTGG

CCCGTTGTCAGGCAACGTGGCGTGGTGTGCACTGTGTTTGCTGACGCAACCCCCACTGGT

TGGGGCATTGCCACCACCTGTCAGCTCCTTTCCGGGACTTTCGCTTTCCCCCTCCCTATT

GCCACGGCGGAACTCATCGCCGCCTGCCTTGCCCGCTGCTGGACAGGGGCTCGGCTGTTG

GGCACTGACAATTCCGTGGTGTTGTCGGGGAAGCTGACGTCCTTTCCATGGCTGCTCGCC

TGTGTTGCCACCTGGATTCTGCGCGGGACGTCCTTCTGCTACGTCCCTTCGGCCCTCAAT

CCAGCGGACCTTCCTTCCCGCGGCCTGCTGCCGGCTCTGCGGCCTCTTCCGCGTCTTCGC

CTTCGCCCTCAGACGAGTCGGATCTCCCTTTGGGCCGCCTCCCCGCATCGGGAATTCCCG

CGGTTCGAATTCTACCGGGTAGGGGAGGCGCTTTTCCCAAGGCAGTCTGGAGCATGCGCT

TTAGCAGCCCCGCTGGGCACTTGGCGCTACACAAGTGGCCTCTGGCCTCGCACACATTCC

ACATCCACCGGTAGGCGCCAACCGGCTCCGTTCTTTGGTGGCCCCTTCGCGCCACCTTCT

ACTCCTCCCCTAGTCAGGAAGTTCCCCCCCGCCCCGCAGCTCGCGTCGTGCAGGACGTGA

CAAATGGAAGTAGCACGTCTCACTAGTCTCGTGCAGATGGACAGCACCGCTGAGCAATGG

AAGCGGGTAGGCCTTTGGGGCAGCGGCCAATAGCAGCTTTGCTCCTTCGCTTTCTGGGCT

CAGAGGCTGGGAAGGGGTGGGTCCGGGGGCGGGCTCAGGGGCGGGCTCAGGGGCGGGGCG

GGCGCCCGAAGGTCCTCCGGAGGCCCGGCATTCTGCACGCTTCAAAAGCGCACGTCTGCC

GCGCTGTTCTCCTCTTCCTCATCTCCGGGCCTTTCGACCTCACGTGCGCATGATTGAACA

AGATGGATTGCACGCAGGTTCTCCGGCCGCTTGGGTGGAGAGGCTATTCGGCTATGACTG

GGCACAACAGACAATCGGCTGCTCTGATGCCGCCGTGTTCCGGCTGTCAGCGCAGGGGCG

CCCGGTTCTTTTTGTCAAGACCGACCTGTCCGGTGCCCTGAATGAACTGCAAGACGAGGC

AGCGCGGCTATCGTGGCTGGCCACGACGGGCGTTCCTTGCGCAGCTGTGCTCGACGTTGT

CACTGAAGCGGGAAGGGACTGGCTGCTATTGGGCGAAGTGCCGGGGCAGGATCTCCTGTC

ATCTCACCTTGCTCCTGCCGAGAAAGTATCCATCATGGCTGATGCAATGCGGCGGCTGCA

TACGCTTGATCCGGCTACCTGCCCATTCGACCACCAAGCGAAACATCGCATCGAGCGAGC

ACGTACTCGGATGGAAGCCGGTCTTGTCGATCAGGATGATCTGGACGAAGAGCATCAGGG

GCTCGCGCCAGCCGAACTGTTCGCCAGGCTCAAGGCGAGCATGCCCGACGGCGAGGATCT

CGTCGTGACCCATGGCGATGCCTGCTTGCCGAATATCATGGTGGAAAATGGCCGCTTTTC

TGGATTCATCGACTGTGGCCGGCTGGGTGTGGCGGACCGCTATCAGGACATAGCGTTGGC

TACCCGTGATATTGCTGAAGAGCTTGGCGGCGAATGGGCTGACCGCTTCCTCGTGCTTTA

CGGTATCGCCGCTCCCGATTCGCAGCGCATCGCCTTCTATCGCCTTCTTGACGAGTTCTT

CTGAGCGGGACTCTGGGTACCTTTAAGACCAATGACTTACAAGGCAGCTGTAGATCTTAG

CCACTTTTTAAAAGAAAAGGGGGGACTGGAAGGGCTAATTCACTCCCAACGAAGACAAGA

TCTGCTTTTTGCTTGTACTGGGTCTCTCTGGTTAGACCAGATCTGAGCCTGGGAGCTCTC

TGGCTAACTAGGGAACCCACTGCTTAAGCCTCAATAAAGCTTGCCTTGAGTGCTTCAAGT

AGTGTGTGCCCGTCTGTTGTGTGACTCTGGTAACTAGAGATCCCTCAGACCCTTTTAGTC

AGTGTGGAAAATCTCTAGCAGTAGTAGTTCATGTCATCTTATTATTCAGTATTTATAACT

TGCAAAGAAATGAATATCAGAGAGTGAGAGGAACTTGTTTATTGCAGCTTATAATGGTTA

CAAATAAAGCAATAGCATCACAAATTTCACAAATAAAGCATTTTTTTCACTGCATTCTAG

TTGTGGTTTGTCCAAACTCATCAATGTATCTTATCATGTCTGGCTCTAGCTATCCCGCCC

CTAACTCCGCCCATCCCGCCCCTAACTCCGCCCAGTTCCGCCCATTCTCCGCCCCATGGC

TGACTAATTTTTTTTATTTATGCAGAGGCCGAGGCCGCCTCGGCCTCTGAGCTATTCCAG

AAGTAGTGAGGAGGCTTTTTTGGAGGCCTAGGGACGTACCCAATTCGCCCTATAGTGAGT

CGTATTACGCGCGCTCACTGGCCGTCGTTTTACAACGTCGTGACTGGGAAAACCCTGGCG

TTACCCAACTTAATCGCCTTGCAGCACATCCCCCTTTCGCCAGCTGGCGTAATAGCGAAG

AGGCCCGCACCGATCGCCCTTCCCAACAGTTGCGCAGCCTGAATGGCGAATGGGACGCGC

CCTGTAGCGGCGCATTAAGCGCGGCGGGTGTGGTGGTTACGCGCAGCGTGACCGCTACAC

TTGCCAGCGCCCTAGCGCCCGCTCCTTTCGCTTTCTTCCCTTCCTTTCTCGCCACGTTCG

CCGGCTTTCCCCGTCAAGCTCTAAATCGGGGGCTCCCTTTAGGGTTCCGATTTAGTGCTT

TACGGCACCTCGACCCCAAAAAACTTGATTAGGGTGATGGTTCACGTAGTGGGCCATCGC

CCTGATAGACGGTTTTTCGCCCTTTGACGTTGGAGTCCACGTTCTTTAATAGTGGACTCT

TGTTCCAAACTGGAACAACACTCAACCCTATCTCGGTCTATTCTTTTGATTTATAAGGGA

TTTTGCCGATTTCGGCCTATTGGTTAAAAAATGAGCTGATTTAACAAAAATTTAACGCGA

ATTTTAACAAAATATTAACGCTTACAATTTAGGTGGCACTTTTCGGGGAAATGTGCGCGG

AACCCCTATTTGTTTATTTTTCTAAATACATTCAAATATGTATCCGCTCATGAGACAATA

ACCCTGATAAATGCTTCAATAATATTGAAAAAGGAAGAGTATGAGTATTCAACATTTCCG

TGTCGCCCTTATTCCCTTTTTTGCGGCATTTTGCCTTCCTGTTTTTGCTCACCCAGAAAC

GCTGGTGAAAGTAAAAGATGCTGAAGATCAGTTGGGTGCACGAGTGGGTTACATCGAACT

GGATCTCAACAGCGGTAAGATCCTTGAGAGTTTTCGCCCCGAAGAACGTTTTCCAATGAT

GAGCACTTTTAAAGTTCTGCTATGTGGCGCGGTATTATCCCGTATTGACGCCGGGCAAGA

GCAACTCGGTCGCCGCATACACTATTCTCAGAATGACTTGGTTGAGTACTCACCAGTCAC

AGAAAAGCATCTTACGGATGGCATGACAGTAAGAGAATTATGCAGTGCTGCCATAACCAT

GAGTGATAACACTGCGGCCAACTTACTTCTGACAACGATCGGAGGACCGAAGGAGCTAAC

CGCTTTTTTGCACAACATGGGGGATCATGTAACTCGCCTTGATCGTTGGGAACCGGAGCT

GAATGAAGCCATACCAAACGACGAGCGTGACACCACGATGCCTGTAGCAATGGCAACAAC

GTTGCGCAAACTATTAACTGGCGAACTACTTACTCTAGCTTCCCGGCAACAATTAATAGA

CTGGATGGAGGCGGATAAAGTTGCAGGACCACTTCTGCGCTCGGCCCTTCCGGCTGGCTG

GTTTATTGCTGATAAATCTGGAGCCGGTGAGCGTGGGTCTCGCGGTATCATTGCAGCACT

GGGGCCAGATGGTAAGCCCTCCCGTATCGTAGTTATCTACACGACGGGGAGTCAGGCAAC

TATGGATGAACGAAATAGACAGATCGCTGAGATAGGTGCCTCACTGATTAAGCATTGGTA

ACTGTCAGACCAAGTTTACTCATATATACTTTAGATTGATTTAAAACTTCATTTTTAATT

TAAAAGGATCTAGGTGAAGATCCTTTTTGATAATCTCATGACCAAAATCCCTTAACGTGA

GTTTTCGTTCCACTGAGCGTCAGACCCCGTAGAAAAGATCAAAGGATCTTCTTGAGATCC

TTTTTTTCTGCGCGTAATCTGCTGCTTGCAAACAAAAAAACCACCGCTACCAGCGGTGGT

TTGTTTGCCGGATCAAGAGCTACCAACTCTTTTTCCGAAGGTAACTGGCTTCAGCAGAGC

GCAGATACCAAATACTGTTCTTCTAGTGTAGCCGTAGTTAGGCCACCACTTCAAGAACTC

TGTAGCACCGCCTACATACCTCGCTCTGCTAATCCTGTTACCAGTGGCTGCTGCCAGTGG

CGATAAGTCGTGTCTTACCGGGTTGGACTCAAGACGATAGTTACCGGATAAGGCGCAGCG

GTCGGGCTGAACGGGGGGTTCGTGCACACAGCCCAGCTTGGAGCGAACGACCTACACCGA

ACTGAGATACCTACAGCGTGAGCTATGAGAAAGCGCCACGCTTCCCGAAGAGAGAAAGGC

GGACAGGTATCCGGTAAGCGGCAGGGTCGGAACAGGAGAGCGCACGAGGGAGCTTCCAGG

GGGAAACGCCTGGTATCTTTATAGTCCTGTCGGGTTTCGCCACCTCTGACTTGAGCGTCG

ATTTTTGTGATGCTCGTCAGGGGGGCGGAGCCTATGGAAAAACGCCAGCAACGCGGCCTT

TTTACGGTTCCTGGCCTTTTGCTGGCCTTTTGCTCACATGTTCTTTCCTGCGTTATCCCC

TGATTCTGTGGATAACCGTATTACCGCCTTTGAGTGAGCTGATACCGCTCGCCGCAGCCG

AACGACCGAGCGCAGCGAGTCAGTGAGCGAGGAAGCGGAAGAGCGCCCAATACGCAAACC

GCCTCTCCCCGCGCGTTGGCCGATTCATTAATGCAGCTGGCACGACAGGTTTCCCGACTG

GAAAGCGGGCAGTGAGCGCAACGCAATTAATGTGAGTTAGCTCACTCATTAGGCACCCCA

GGCTTTACACTTTATGCTTCCGGCTCGTATGTTGTGTGGAATTGTGAGCGGATAACAATT

TCACACAGGAAACAGCTATGACCATGATTACGCCAAGCGCGCAATTAACCCTCACTAAAG

GGAACAAAAGCTGGAGCTGCAAGCTT

**Supplemental Figure S28**


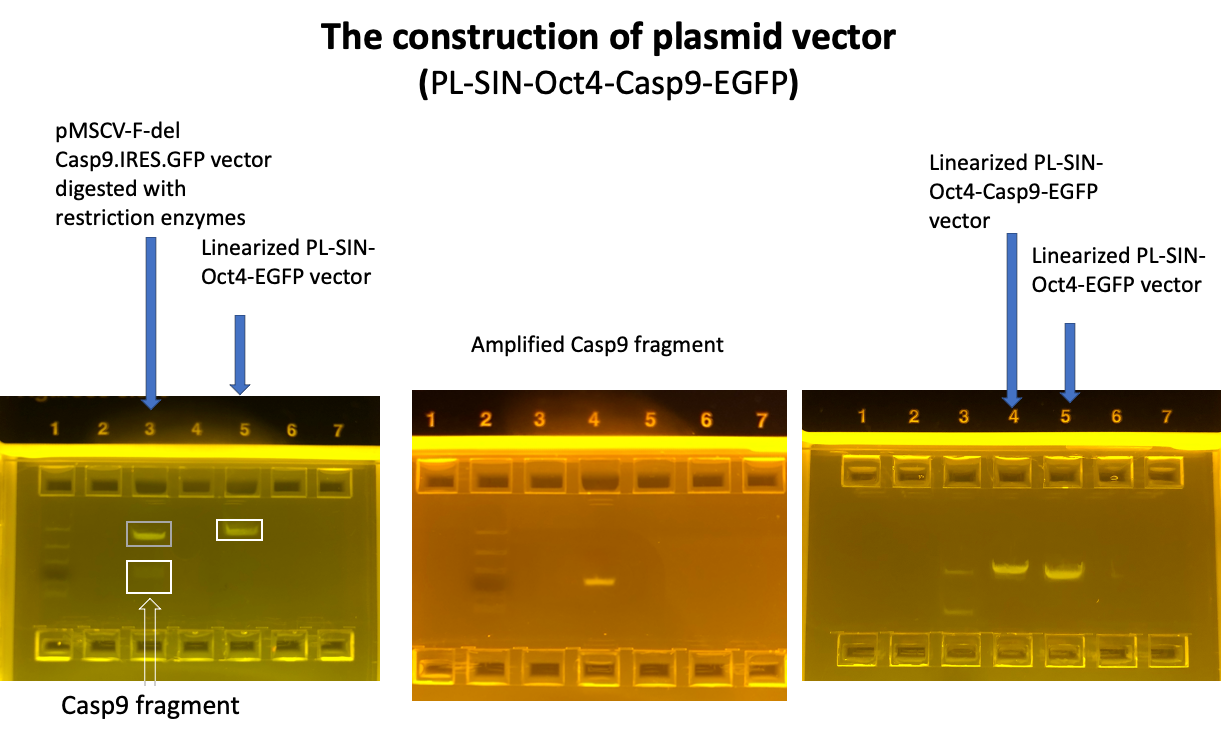
Representative fragment images on the construction of new vector (PL-SIN-Oct4(POU5F1)-Casp9-EGFP).

**Table S1. The details of chemotherapy regimen and evaluation**

| Patient ID |  | Regimen | RECIST*  (6months) | | Tumor volume** | Time for evaluate | Final confirmation | | Prognosis | |  |  |
| --- | --- | --- | --- | --- | --- | --- | --- | --- | --- | --- | --- | --- |
| 759_pt | FOLFIRI+Cmab | | PR | 0.3713 | | 8week | | 133week | | Alive | |  |
| 9311_pt | Xeloda +Bev | | PR | 0.6540 | | 12week | | 68week | | Alive | |  |
| 747_pt | FOLFOXIRI +Bev | | PR | 0.7000 | | 12week | | 40week | | Dead | |  |
| 1M29F_pt | FOLFOX+Bmab | | SD | 0.7550 | | 16week | | 26week | | Dead (non-cancer death) | |  |
| 6524_pt | FOLFOX+Bmab | | SD | 1.1153 | | 24week | | 144 week | | Alive | |  |
| 9516_pt | Cap+Bab | | PD | 1.4477 | | 20week | | 36week | | Alive | |  |
| 25D_pt | FOLFOX+Bmab | | PD | 1.8551 | | 4week | | 8week | | Dead | |  |
| 603_pt | FOLFIRI+Rmab | | PD | 2.0476 | | 12week | | 28week | | Dead | |  |

***** PR-SD: The tumor volume of the time when the tumor became the smallest up to 24 weeks. PD: The tumor volume at the end of the regimen

** When tumor size was measured from the start of anticancer drug treatment

**Table S2. Antibodies lists for flow cytometric analysis**

| **Target** | **Product No.** | **Campany** |
| --- | --- | --- |
| EpCAM | 326808 | BioLegend, San Diego, CA, USA |
| CD133 | 372808 | BioLegend, San Diego, CA, USA |
| CD44 | 338820 | BioLegend, San Diego, CA, USA |
| CD41 | 30370 | BioLegend, San Diego, CA, USA |
| CD45 | 304012 | BioLegend, San Diego, CA, USA |
| LGR5 | 130-100-847 | Miltenyi Biotec, Bergisch Gladbach, Germany |
| POU5F1 | 653712 | BioLegend, San Diego, CA, USA |
| POU5F1 | 653704 | BioLegend, San Diego, CA, USA |

**Table S3. Drug list used in clinical drug assay**

| Compaund name | Diluted solution | MW | ＊Final concentration of assay (μM) | CAS |
| --- | --- | --- | --- | --- |
| 7-ETHYL-10-HYDROXYCAMPTOTHECIN | DMSO | 677.18 | 7.09 | 86639-52-3 |
| Vinorelbine Ditartrate | DW | 1079.11 | 0.74 | 125317-39-7 |
| Paclitaxel | DMSO | 853.91 | 7.87 | 33069-62-4 |
| Docetaxel | DMSO | 861.93 | 2.78 | 114977-28-5 |
| 4-Hydroxycyclophosphamide | DW | 279.1 | 85.99 | 40277-05-2 |
| Amrubicinol (Mixture of Diastereomers) | DW | 519.93 | 2.77 | 186353-53-7 |
| Epirubicin Hydrochloride | DW | 579.98 | 5.52 | 56390-09-1 |
| Doxorubicin Hydrochloride | DW | 579.98 | 4.41 | 25316-40-9 |
| Gemcitabine hydrochloride | DW | 299.66 | 106.79 | 122111-03-9 |
| 5-Fluorouracil | DMSO | 130.08 | 184.50 | 51-21-8 |
| Pemetrexed Disodium Heptahydrate | DW | 597.48 | 26.78 | 357166-29-1 |
| Methotrexate | DW | 454.44 | 264.06 | 59-05-2 |
| Oxaliplatin | DW | 397.29 | 10.47 | 61825-94-3 |
| Cisplatin | DW | 300.05 | 10.66 | 15663-27-1 |
| Cetuximab | DW | 151800 | 0.08 | 205923-56-4 |
| Trastuzumab | DW | 148000 | 0.05 | 180288-69-1 |
| Trastuzumab Emtansine | DW | 151000 | 0.02 | 1018448-65-1 |
| Panitumumab | DW | 147000 | 0.05 | 339177-26-3 |
| Pertuzumab | DW | ― | 16.8 (μg/mL)** | 380610-27-5 |
| Eribulin (mesylate) | DW | 826 | 0.05 | 441045－17－6 |

* The concentration is a pharmacologically calculated value to estimate the concentration in human plasma based on the amount of drug administered clinically.

**Since the molecular weight is not clearly defined, the concentration is given.**Table S4. Primers lists**

| Target gene | Company | Catalog number |
| --- | --- | --- |
| GAPDH | BIO-RAD | qHsaCED0038674 |
| POU5F1 | BIO-RAD | qHsaCED0038334 |
| CASP9 | BIO-RAD | qHsaCED0044476 |
| NKD1 | BIO-RAD | qHsaCID0015242 |
| BAMBI | BIO-RAD | qHsaCID0014107 |
| CTLA4 | BIO-RAD | qHsaCED0003794 |

**Table S5. Antibodies lists and dilution for immunohistochemistry**

| **Target** | **Product No.** | **Campany** | **Dilution** |
| --- | --- | --- | --- |
| CD44 | GTX102111 | Gene Tex, CA,USA | 1:250 |
| CK20 | ab854 | Abcam Inc.,Cambridge, UK | 1:100 |
| MUC2 | ab133555 | Abcam Inc.,Cambridge, UK | 1:100 |
| Chromogranin A | ab68271 | Abcam Inc.,Cambridge, UK | 1:50 |
| CACFD1  (Flower) | AA41-130 | Antibodies-online GmbH, Aachen,Germany | 1:100 |

**Table S6. Antibodies lists and dilution for immunocytochemistry**

| **Target** | **Product No.** | **Campany** | **Dilution** |
| --- | --- | --- | --- |
| POU5F1 | ab181557 | Abcam Inc.,Cambridge, UK | 1:125 |
| CTLA4 | ab19792 | Abcam Inc.,Cambridge, UK | 1:50 |
| CACFD1  (Flower) | AA41-130 | Antibodies-online GmbH, Aachen,Germany | 1:100 |
| Cytokeratin 20 | ab854 | Abcam Inc.,Cambridge, UK | 1:100 |
| EpCAM | 36746 | CST, Danvers, MA, USA | 1:100 |
| Vimentin | NBP2-44833 | Novus Biologicals, LLC, CO,USA | 1ug/mL |
| Rabbit IgG_AF488 | A11008 | Thermo Fisher Scientific, Waltham, MA, USA | 1:2000 |
| Mouse IgG_AF594 | A11005 | Thermo Fisher Scientific, Waltham, MA, USA | 1:2000 |
| Rabbit IgG_AF647 | A21244 | Thermo Fisher Scientific, Waltham, MA, USA | 1:2000 |
