# Supplementary material for: Metastases and treatment-resistant lineages in patient-derived cancer cells of colorectal cancer
Source: Commun Biol. 2023 Nov 24;6:1191. doi: 10.1038/s42003-023-05562-y (PMC10667365; doi:10.1038/s42003-023-05562-y)
Supplement: Supplementary file 2 — Description of additional supplementary files [file 42003_2023_5562_MOESM2_ESM.docx]

Description of Additional Supplementary Files

**File name:** Supplementary Data 1

**Description:** Top 100 gene sets significantly enriched in poor-response 2DOs than good response 2Dos.

**File name:** Supplementary Data 2

**Description:** Characteristic genes in each cluster

**File name:** Supplementary Movie 1

**Description:** The source data behind the snapshots of figure 3h.
